# SARS-CoV-2 infection and replication in human gastric organoids

**DOI:** 10.1038/s41467-021-26762-2

**Published:** 2021-11-16

**Authors:** Giovanni Giuseppe Giobbe, Francesco Bonfante, Brendan C. Jones, Onelia Gagliano, Camilla Luni, Elisa Zambaiti, Silvia Perin, Cecilia Laterza, Georg Busslinger, Hannah Stuart, Matteo Pagliari, Alessio Bortolami, Eva Mazzetto, Anna Manfredi, Chiara Colantuono, Lucio Di Filippo, Alessandro Filippo Pellegata, Valentina Panzarin, Nikhil Thapar, Vivian Sze Wing Li, Simon Eaton, Davide Cacchiarelli, Hans Clevers, Nicola Elvassore, Paolo De Coppi

**Affiliations:** 1 Stem Cell and Regenerative Medicine Section, GOS Institute of Child Health, University College London, London, UK; 2 Lab. of Experimental Animal Models, Division of Comparative Biomedical Sciences, Istituto Zooprofilattico Sperimentale delle Venezie, Legnaro, Italy; 3 Veneto Institute of Molecular Medicine (VIMM), Padova, Italy; 4 Shanghai Institute for Advanced Immunochemical Studies (SIAIS), ShanghaiTech University, Shanghai, China; 5 Dept. Women’s and Children’s Health, University of Padova, Padova, Italy; 6 Oncode Institute, Hubrecht Institute, Royal Netherlands Academy of Arts and Sciences (KNAW) and University Medical Center (UMC) Utrecht, Utrecht, Netherlands; 7 Telethon Institute of Genetics and Medicine (TIGEM), Armenise/Harvard Laboratory of Integrative Genomics, Pozzuoli, Italy; 8 Next Generation Diagnostic srl, Pozzuoli, Italy; 9 Gastroenterology, Hepatology and Liver Transplant, Queensland Children’s Hospital, Brisbane, Australia; 10 Stem Cell and Cancer Biology Lab, the Francis Crick Institute, London, UK; 11 Department of Translational Medicine, University of Naples Federico II, Naples, Italy; 12 Princess Máxima Center (PMC) for Pediatric Oncology, Utrecht, Netherlands; 13 Dept. of Industrial Engineering, University of Padova, Padova, Italy; 14 Dept. of Specialist Neonatal and Paediatric Surgery, Great Ormond Street Hospital, London, UK

**Keywords:** Adult stem cells, Gastrointestinal models, SARS-CoV-2, Viral infection

## Abstract

COVID-19 typically manifests as a respiratory illness, but several clinical reports have described gastrointestinal symptoms. This is particularly true in children in whom gastrointestinal symptoms are frequent and viral shedding outlasts viral clearance from the respiratory system. These observations raise the question of whether the virus can replicate within the stomach. Here we generate gastric organoids from fetal, pediatric, and adult biopsies as in vitro models of SARS-CoV-2 infection. To facilitate infection, we induce reverse polarity in the gastric organoids. We find that the pediatric and late fetal gastric organoids are susceptible to infection with SARS-CoV-2, while viral replication is significantly lower in undifferentiated organoids of early fetal and adult origin. We demonstrate that adult gastric organoids are more susceptible to infection following differentiation. We perform transcriptomic analysis to reveal a moderate innate antiviral response and a lack of differentially expressed genes belonging to the interferon family. Collectively, we show that the virus can efficiently infect the gastric epithelium, suggesting that the stomach might have an active role in fecal-oral SARS-CoV-2 transmission.

## Abstract (web summary)

Several clinical reports have described gastrointestinal symptoms for COVID-19, though whether the virus can replicate within the stomach remains unclear. Here the authors generate gastric organoids from human biopsies and show that the virus can efficiently infect gastric epithelium, suggesting that the stomach might have an active role in fecal-oral transmission.

## Introduction

Severe acute respiratory syndrome coronavirus 2 (SARS-CoV-2) is responsible for a pandemic that has proven catastrophic, partly due to the lack of immunity in the human population and the range of pathological features associated with infection, including severe and often life-threatening respiratory syndromes^1^ causing major health, social, and economic consequences. Understanding the pathogenesis and the mechanisms for transmission is of utmost importance. A growing body of literature suggests that replication at the level of the gastrointestinal (GI) tract not only occurs in a large proportion of cases^2,3^, but it also extends the overall duration of shedding, after viral clearance from the respiratory tract has occurred^4^. Moreover, SARS-CoV-2 has been detected in adult stool samples^5^ and in air samples of patients’ toilet areas^6^. Interestingly, infected children have been shown to be particularly prone to develop GI symptoms which can be moderate-to-severe, mimicking, in a minority of cases, symptoms of appendicitis^7^, and leading to intensive care unit (ICU) admission. This evidence, together with the demonstration of a high receptor density at the level of the oral cavity^8^, raise important questions regarding oral infection and fecal-oral transmission and their dependency on age.

Defining the role of the GI tract in respect to the infection may also help understanding the possibility of vertical transmission since amniotic fluid is swallowed by the fetus and viable virus has been isolated from the placenta of an affected woman^9^. Investigating the susceptibility in vitro of the GI tract during different fetal developmental stages will help to elucidate a potential mechanism for vertical transmission. While samples from affected mothers have so far failed to prove that amniotic fluid, cord blood, and breast milk contain SARS-CoV-2^10^, a paucity of data prevent firm conclusions on the role of COVID-19 on intrauterine vertical transmission^11–13^.

Reliable human in vitro GI model systems that faithfully reproduce infection dynamics and disease mechanisms will prove key to advance our understanding of SARS-CoV-2 replication and pathology in the GI tract. In particular, we lack fundamental information regarding which region of the GI system is the target of replication and prolonged shedding of SARS-CoV-2 in both pediatric and adult patients. Organoids have proven a useful tool for in vitro disease modeling and for studying infectious pathogens, particularly of the GI tract^14^. Recent studies have demonstrated how SARS-CoV-2 can efficiently infect human intestinal enteroids^15,16^, providing evidence in support of the hypothesis that SARS-CoV-2  can be transmitted via the fecal-oral route. However, it remains to be elucidated whether access to the duodenum depends on passive transport of infected oral fluids across the stomach, or on active viral replication in the gastric mucosa^17^. Human gastric organoids derived from adult patients and induced pluripotent stem cells have proved to be instrumental for the generation of reliable in vitro models for the characterization of infectious agents^18–20^. Organoid derivation from human fetal organs has been shown for the intestine^21^, liver^22^, and pancreas^23^, but not as yet for the stomach.

This work aims to unravel the susceptibility of the stomach mucosa to SARS-CoV-2 infection through the development of an innovative expandable in vitro model that faithfully reproduces the gastric microenvironment. A deeper understanding of the susceptibility of the human stomach to SARS-CoV-2 infection and replication could lay the foundations for the development of therapeutic options to reduce gastrointestinal infection. In this work, we describe the derivation of proliferative progenitors from human fetal stomach and their expansion in vitro as organoids, and subsequent detailed characterization and comparison to developmental stage matched tissue of origin, We then optimize the gastric organoid system for modeling infection by reversing organoid polarity, allowing us to provide insight into the ability of SARS-CoV-2 to infect an organoid-based model of the gastric epithelium at different developmental stages of life.

## Results

### Defined stages are present during early and late human gastric development

**Fig. 1:**
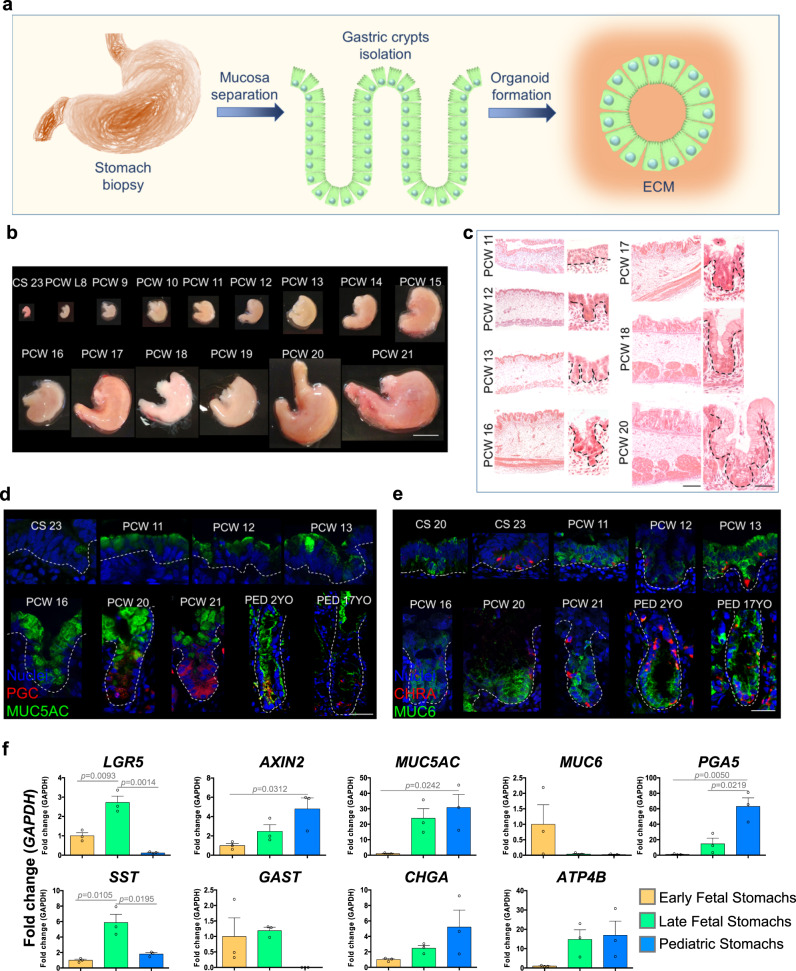
Human fetal stomach characterization. **a** Schematic of the fetal and pediatric stomachs isolation, characterization, and gastric organoid derivation. **b** Isolated human whole stomachs from terminated pregnancies, from Carnegie stage 23 (mid-week 8) to post conception week (PCW) 21. Scale bar 1 cm. **c** Hematoxylin and Eosin staining of paraffin-embedded stomach sections from PCW 11 to PCW 20. Scale bars 100 μm in lower magnification (left) and 20 μm in higher gland magnification (right). **d** Immunofluorescence panel showing mucin 5AC (MUC5AC) in green, pepsinogen C (PGC) in red and nuclei in blue (Hoechst). Scale bar 20 μm. **e** Immunofluorescence panel showing mucin 6 (MUC6) in green, chromogranin A (CHRA) in red and nuclei in blue (Hoechst). Scale bar 20 μm. **f** Real-time PCR analysis of early fetal, late fetal, and pediatric stomachs. Relative fold change to GAPDH. Mean ± SEM (*n* = 3). Black circles on the bar charts represent single biological replicates. Ordinary one-way ANOVA.

Organoids are organized three dimensional structures that can be grown from stem cells found in adult and fetal tissues. In order to derive an in vitro gastric model of fetal origin, we first characterized the tissues isolated from human fetuses and compared them to gastric mucosa obtained from pediatric patients undergoing surgery (Fig. 1a). Developing stomach structures are shown in Fig. 1b from Carnegie stage (CS) 23 (corresponding to mid-week 8) to post conception week (PCW) 21. Gastric glands start to invaginate between PCW 11 and PCW 12 and form a clearly defined  gland at around PCW 20 (Fig. 1c). We characterized the appearance of gastric markers during stomach development. Mucin 5AC positive pit mucous cells were evident at PCW 11, while pepsinogen C (marking chief cells) started to emerge at around PCW 20 (Fig. 1d). Mucin 6, a gland mucous cell marker, was constitutively expressed from early week 8 (CS 20), together with enteroendocrine cells marked by chromogranin A that were present from mid-week 8 (CS 23) (Fig. 1e). We then defined three distinct groups of gastric epithelial tissues based on gland maturity: (1) early fetal stomachs from PCW 8 to PCW 15; (2) late fetal stomachs from PCW 17 to PCW 21; and (3) pediatric stomachs. Real-time quantitative PCR (qPCR) was performed on gastric tissues obtained from these three groups to examine the gene expression changes of stem cell and mature cell markers. A significant correlation between developmental stage and mRNA expression was observed for *AXIN2*, mucin 5AC (*MUC5AC*), pepsinogen A5 (*PGA5*), with a similar trend for chromogranin A (*CHGA*) and ATPase H+/K+transporting subunit beta (*ATP4B*) (Fig. 1f). On the other hand, expression of leucine-rich repeat-containing G-protein coupled receptor 5 (*LGR5*) and somatostatin (*SST*) were significantly higher in the late fetal stomachs.

### Gastric organoids can be derived from human tissue across different developmental stages

**Fig. 2:**
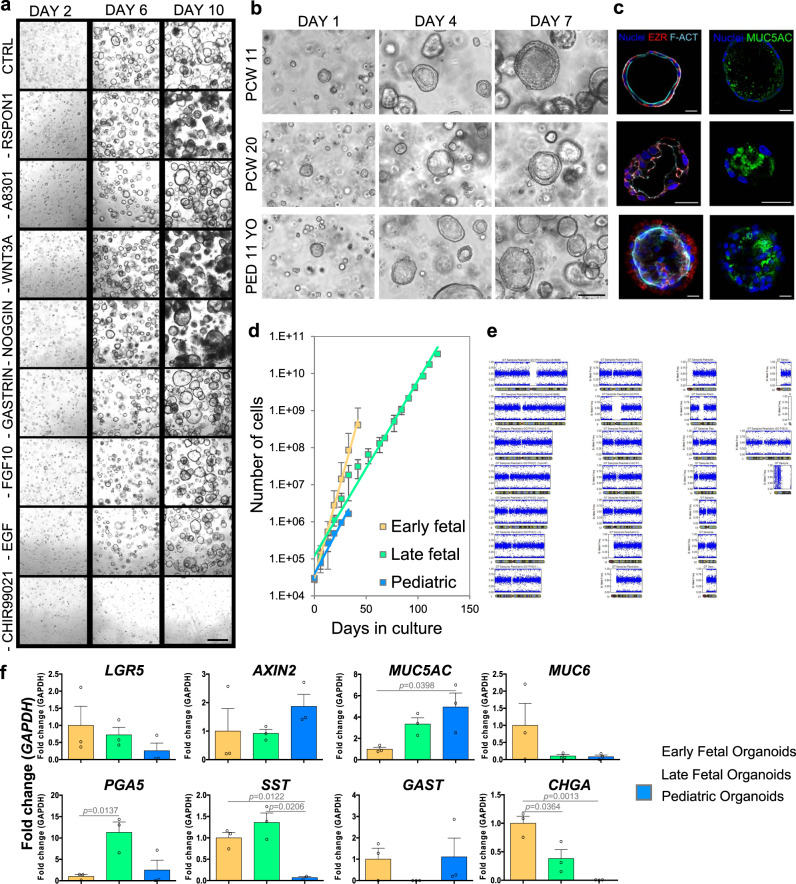
Fetal gastric organoid derivation, expansion, and characterization. **a** Selective withdrawal of gastric organoid cytokines from control (CTRL) complete medium, in CS 23 (mid-week 8) organoid line split at single cells at passage 7. Scale bar 400 μm. **b** Bright field images of representative organoid line for each of the 3 stages, showing the formation of spherical organoids within 7 days starting from single cells. Scale bar 100 μm. **c** Immunofluorescence panel showing ezrin (EZR) in red, f-actin (F-ACT) in cyan, mucin 5AC (MUC5AC) in green, and nuclei in blue (Hoechst). Scale bars 25 μm. **d** Cumulative cell counts during days of culture. Mean ± SD (*n* = 4 biological replicates for early and late fetal, *n* = 3 for pediatric organoids). **e** Single-nucleotide polymorphism (SNP) array for pediatric organoids. Representative image of the chromosome viewer and allele frequency. **f** Real-time PCR analysis of early fetal, late fetal, and pediatric gastric organoids. Relative fold change to GAPDH. Mean ± SEM (*n* = 3). Black circles on the bar charts represent single biological replicates. Ordinary one-way ANOVA.

Following gastric tissue characterization, we efficiently extracted glands from stomach biopsies utilizing chelating buffers and mechanical stress. Adult organoid lines were already available in the lab^19,24,25^, therefore we optimized the protocol for organoids of fetal and pediatric origin. To improve compatibility with subsequent clinical application of this organoid system, isolated cells were expanded in a chemically defined medium, without the use of animal serum or conditioned media. Each gastric cytokine, based on previous work^19,26^ was screened and selectively removed from the organoids split to single cells and grown for 10 days to allow clonal organoid formation. While R-spondin 1, wnt-3A and noggin withdrawal led to more unhealthy organoids at day 10, CHIR99021 (GSK-3 inhibitor) proved to be essential in the formation of fetal gastric organoids starting from single cells (Fig. 2a). No medium-related difference was observed among multiple organoid stages. We then performed isolation of several gastric organoid lines (Supplementary Table 1). The isolation protocol proved to be highly efficient, and we obtained a biobank composed of multiple developmental stages. Expanding organoids were stained for the epithelial marker ezrin (EZR) and luminal polarized f-actin Fig. 2c. MUC5AC was present on the luminal side of the organoids of all stages, with a relatively lower expression in the early PCW 11 (Fig. 2c). Organoids were expanded and counted for several months, showing higher rate of expansion for earlier fetal stages (Fig. 2d). No plateau was reached in any of the curves even after several months, showing the possibility to obtain stable gastric organoid lines of fetal origin (Supplementary Fig. 1a). After weekly passaging for more than 10 weeks, we further characterized the organoid lines to evaluate genomic stability. Single-nucleotide polymorphism (SNP) arrays on early fetal, late fetal, and pediatric organoids showed no chromosomal duplications, no large deletions, nor other karyotype aberrations, demonstrating the organoids are genetically stable after prolonged in vitro culture (Fig. 2e, Supplementary Fig. 1b). Real-time PCR was performed on organoids grouped in early fetal (CS 23 to PCW 11), late fetal (PCW 18 to PCW 20) and pediatric. Stem cell gland markers *LGR5* and *AXIN2* were expressed in these organoids, indicating the presence of proliferating cells. *MUC5AC*, *MUC6*, *SST*, and *AXIN2* showed comparable pattern of expression between stomachs and organoids (Fig. 1f). On the other hand, *LGR5*, *PGA5*, *GAST*, and *CHGA* showed a different pattern of expression, while transcript expression of proton pump transporter *ATP4B*, responsible for gastric acid secretion, was lost in the organoid model (Fig. 2f).

### Gastric organoids of fetal and pediatric origin preserve only partial transcriptional features of the tissues of origin

**Fig. 3:**
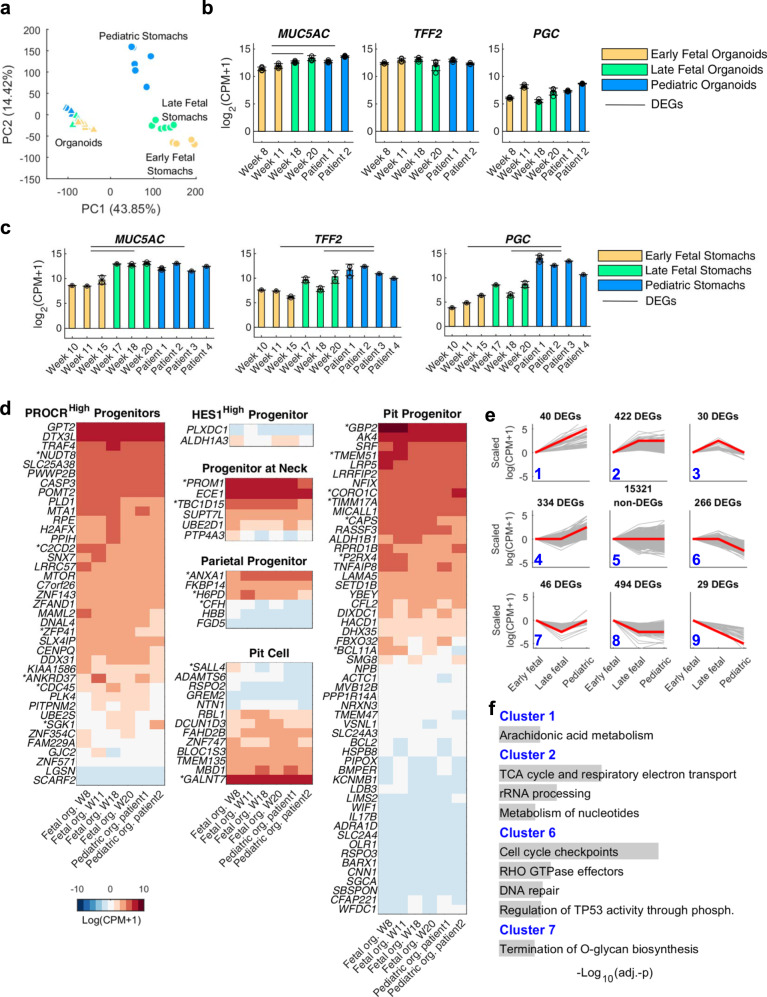
Transcriptomic characterization of gastric tissues and tissue-derived organoids from different stages of development. **a** Principal component analysis (PCA) of RNA-sequencing samples from organoids (triangles) and fetal gastric epithelial tissues (circles) at different stages of development. Edge color identifies the biological replicates or patients within the same group of samples. Stages of fetal tissues or derived gastric organoids: early fetal (PCW 8–15), late fetal (week 17–20), and pediatric. **b**, **c** Expression of typical gastric markers in organoids (**b**) and gastric epithelium (**c**) at different stages of development. Black circles indicate single data points. Black error bar: mean ± SD (*n* = 4 for organoids biological replicates, *n* = 3 for biological replicates tissues). CPM: counts per million. Horizontal lines above the bar plots highlight DEGs between the indicated conditions. **d** Results of hierarchical clustering of organoid gene expression. The selected genes were previously shown to define the six stomach cellular subtypes identified in Gao et al.^28^ by single-cell RNA-seq, as indicated on top of each plot. Genes whose name is preceded by an asterisk (*) are differentially expressed genes (DEGs) between any pair of conditions. **e** Pseudo-temporal profiles of gene expression according to differential expression analysis. DEGs were clustered according to a flat, increasing, or decreasing profile between pairs of time points. Data were scaled respect to the first time point. Red lines: average profile of each cluster. Gray lines: profile of each gene in the cluster. Blue digits: cluster identification number. **f** Selected categories from Reactome enrichment analysis of genes in the clusters displayed in **e**. Gray bars: graphical representation of the values of the adjusted *p*-value (always <0.01), corrected by Benjamini–Hochberg method, from the right-sided hypergeometric test. Full results are displayed in the Supplementary Information.

Next, we characterized the transcriptomics of gastric epithelial tissues and gastric-derived organoids, at three developmental stages. While organoids of adult origin had been previously characterized^27^, we further performed ad hoc RNA-seq characterization of fetal and pediatric stages. Principal component analysis (PCA) showed smaller heterogeneity in the organoid groups derived at different stages of fetal and pediatric development with respect to the primary tissues analyzed at the same stages, which may also include some heterogeneity from the surrounding cells as a result of the isolation procedure (Fig. 3a). When PCA was performed including only organoid samples, the overall variability due to the different developmental stage was comparable to that between biological replicates within the same group (Supplementary Fig. 2a). This analysis suggests that transcriptional differences related to the developmental stage of the tissue of origin could be more subtle than those captured at PCA level. We then analyzed the expression of typical gastric markers in organoids derived from tissues at different stages^28^. The only differentially expressed gene (DEG) was *MUC5AC*, which was more highly expressed in organoids from tissues at later developmental stages (Fig. 3b and Supplementary Fig. 2b), confirming the qPCR results above (Fig. 2f). We did not observe processes of “intestinalization” of the organoids in culture, as *CDX2* expression was negligible (Supplementary Fig. 2c). Consistent with the qPCR results in Fig. 2f, expression of *ATP4A* and *ATP4B* proton transporters were not detectable in the RNA-seq, confirming the absence of the parietal cells in the organoids (Supplementary Fig. 2d). On the other hand, most putative genes identifying gastric gland stem cells (*SOX9, OLFM4, PROCR, MKI67, TACSTD2*) were expressed at all developmental stages (Supplementary Fig. 2e). By differential expression analysis, we selected all genes that showed a consistently decreasing or increasing pattern from early fetal to late fetal to pediatric stages and identified multiple genes that had a reproducible trend both in stomach tissues and in organoids (Supplementary Fig. 2f, g and Supplementary Data 1). RNA-seq analysis on the gastric primary tissues showed a significant increase in transcript levels of the functional markers along the developmental stage (Fig. 3c), confirming that the temporal trend shown by PCA (Fig. 3a) is related to specific gastric developmental stages. When we performed hierarchical clustering analyses of the previously reported genes representing the six stomach cellular subtypes^28^, most of these genes from the analysis were not DEGs (Fig. 3d). Indeed, these six cell types are known to be all co-present at different stages of embryo development from PCW 7 to 25^28^. Furthermore, we clustered DEGs between pair of conditions to reproduce a pseudo-temporal profile between the three developmental stages considered (Fig. 3e). We highlighted in Fig. 3f the results of a pathway enrichment analysis from selected clusters that displayed gastric-related functions. Full results are reported in Supplementary Data 2.

### Reverse polarity in organoids facilitates expression of ACE2 and TMPRSS2 on the external surface

**Fig. 4:**
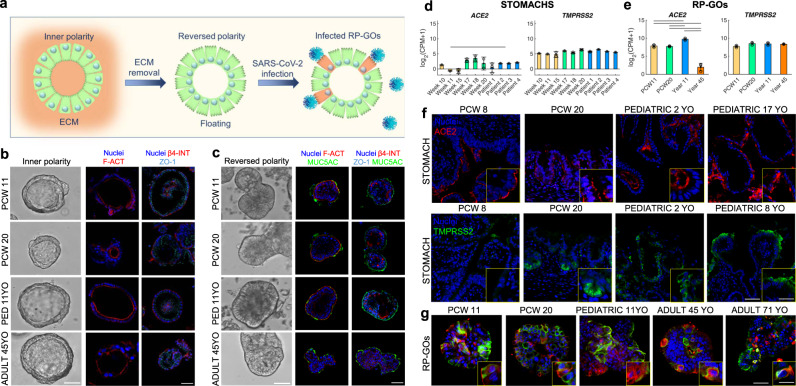
Reverse polarity organoids for efficient SARS-CoV-2 infection. **a** Schematic showing the generation of reverse polarity gastric organoids (RP-GOs). **b** Gastric organoids with normal (inner) polarity and large lumen. Immunofluorescence panel showing f-actin (F-ACT) in red, zonula occludens-1 (ZO-1) in violet, β-4 integrin (β-4 INT) in red, and nuclei in blue (Hoechst). Scale bar 50 μm. **c** RP-GOs showing an almost absent lumen. Immunofluorescence panel showing f-actin (F-ACT) in red, zonula occludens-1 (ZO-1) in violet, β-4 integrin (β-4 INT) in red, mucin 5AC (MUC5AC) in green and nuclei in blue (Hoechst). Scale bar 50 μm. **d**, **e** SARS-CoV-2 receptors, angiotensin-converting enzyme 2 (ACE2) and transmembrane protease serine 2 (TMPRSS2) absolute RNA-seq expression in gastric tissues (left) and gastric organoids (right). Mean ± SD (*n* = 3 for tissues biological replicates, *n* = 3 for organoids). CPM count per million. Horizontal lines above the bar plots highlight DEGs between the indicated conditions. **f** Immunofluorescence panel showing ACE2 in red, TMPRSS2 in green and nuclei in blue (Hoechst) in fetal and pediatric stomach biopsies. Scale Bar 50 µm (main figures) and 30 µm (enlargement). **g** Immunofluorescence panel showing ACE2 in red, TMPRSS2 in green, and nuclei in blue (Hoechst) in fetal, pediatric, and adult gastric organoids with reverse polarity. Scale bar 30 μm (main figures) and 10 µm (enlargement).

In order to validate gastric organoids as functional in vitro models of SARS-CoV-2 infection and replication, we optimized the culture condition for viral infection in a 3D system (Fig. 4a). Standard organoids of endodermal organs have a luminal polarity facing the internal portion of the structure, with an apical (inner) f-actin and zonula occludens-1 (ZO-1), and basal (external) lamina marked by β-4 integrin (β4-INT) (Fig. 4b and Supplementary Fig. 3a). To maximize the efficiency of infection and the effective quantification of the released viral progeny, we reverted the polarity of gastric organoids^29^ to expose the apical side of the cells on the outer surface of the organoids. Organoids were removed from the surrounding extracellular matrix and cultured in suspension for 3 days, resulting in the exposure of the apical f-actin on the outer surface, accompanied with MUC5AC secretion externally (Fig. 4c). Conversely, ZO-1 and β4-INT expression was inverted compared to standard organoids in Fig. 4b. Full 3D deconvolution images of reverse organoids are shown in Supplementary Fig. 3b. PCA analysis on organoid RNA sequencing data showed similar clustering among the different stages of normal polarity organoids (Supplementary Fig. 2a) and reverse-polarity organoids (RP-GOs) (Supplementary Fig. 3c).

We then analyzed the absolute expression of *ACE2* and transmembrane protease serine 2 (TMPRSS2) in our gastric models of fetal and pediatric origin and in the tissues of origin. Unfortunately, native adult gastric tissues were not available at the time of these analyses for such a comparison. RNA-seq data analysis showed that expression of *ACE2* was significantly lower in early fetal stomachs compared to the pediatric ones, while late fetal samples’ higher variability prevented us from drawing a conclusion. On the other hand, *TMPRSS2* mRNA expression was consistently high in all stomach samples irrespective of stages (Fig. 4d). In RP-GOs, the expression profiles for *TMPRSS2* were similar across all stages, while for *ACE2*, RP-GOs of adult and pediatric origin recorded the lowest and the highest levels, respectively. Organoids of fetal origin had similar intermediate counts that were significantly lower than the pediatric ones (Fig. 4e). While presence of ACE2 and TMPRSS2 proteins had already been shown for the adult stomach^30^, we confirmed their expression by immunofluorescence staining in fetal and pediatric gastric biopsies (Fig. 4f) and proved their co-presence in all of the RP-GOs derived at PCW11, PCW 20, pediatric, and adult stages (Fig. 4g).

### Human gastric organoids are susceptible to SARS-CoV-2 infection

**Fig. 5:**
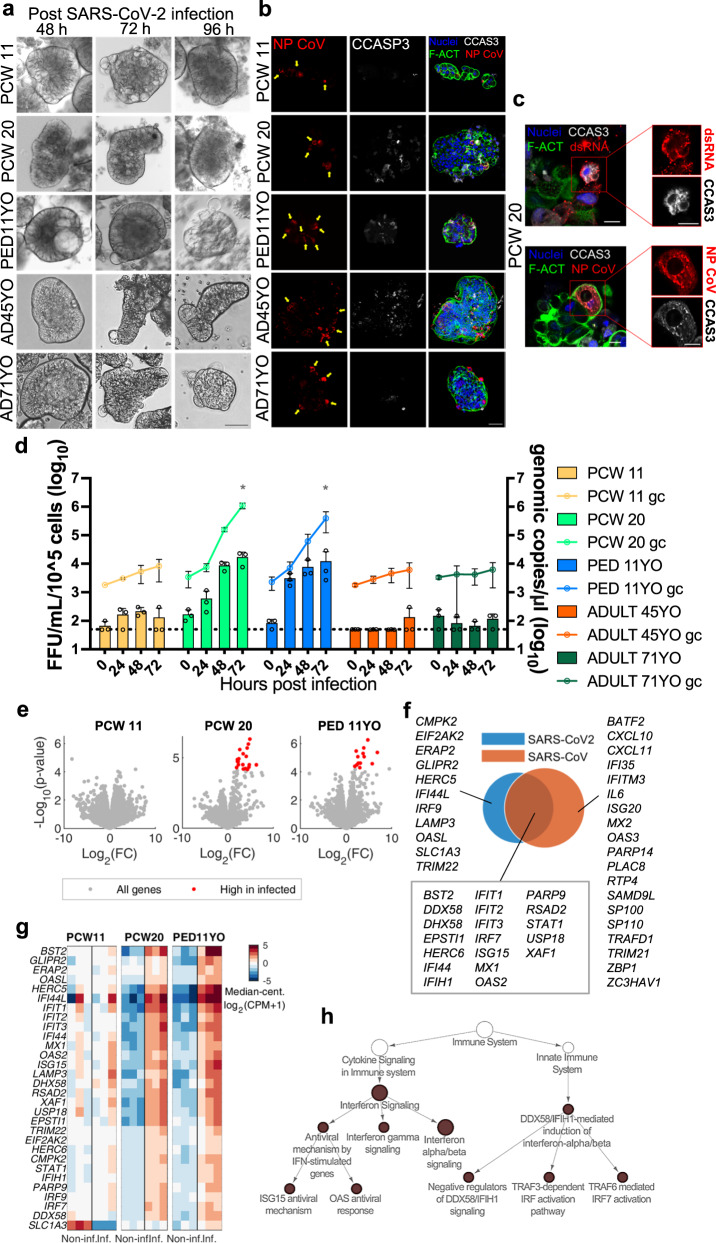
SARS-CoV-2 infection of undifferentiated reverse polarity organoids human gastric organoids. **a** Bright field images of RP-GOs infected with pediatric patient-derived SARS-CoV-2 for 2 h, and acquired at 48, 72 and 96 h post-infection. Scale bar 50 μm. **b** Infected cells in RP-GOs fixed at 96 h post-infection. Immunofluorescence panel showing SARS-CoV-2 Nucleocapsid Antibody (NP CoV) in red, marking infected cells (yellow arrows), cleaved caspase 3 (CCAS3) in white, marking apoptotic cells, f-actin (F-ACT) in green, and nuclei in blue (Hoechst). Image is representative of at least five similar organoid images. Scale bar 50 μm. **c** Infected cell details in RP-GOs fixed at 96 h post-infection. Immunofluorescence panel showing viral double strand RNA J2 (dsRNA) in red, SARS-CoV-2 Nucleocapsid Antibody (NP CoV) in red, cleaved caspase 3 (CCAS3) in white, f-actin (F-ACT) in green, and nuclei in blue (Hoechst). Scale bars 10 μm. **d** Graph of SARS-CoV-2 replication in fetal, pediatric, and adult undifferentiated RP-GOs. Live virus yield was titrated by FFA on Vero E6 cells of culture supernatants collected at 0, 24, 48 and 72 h after infection with SARS-CoV-2 (MOI of 0.5). The dotted line indicates the lower limit of detection. Black circles indicate single data points. Mean ± SD (*n* = 3 experimental replicates). Two-way ANOVA correction for multiple comparison using statistical hypothesis testing: Tukey test 0.002; *p*-value < 0,001. **e**–**h** RNA-seq analysis highlights differential response to infection in organoids derived from different developmental stages. **e** Volcano plots indicating DEG genes of non-infected vs. infected sample comparisons at each developmental stage (PCW 11, PCW 20, 11-years-old pediatric). **f** Venn diagram of DEGs that responded differently to the infection in any developmental stage in this study and DEGs identified in a literature survey of transcriptomic data on SARS-CoV transfected samples^50^. **g** Hierarchical clustering of DEG genes included in **f**, data were median-centered for each pair of non-infected and infected conditions. **h** Results from an enrichment analysis within Reactome database of DEGs highlighted in **e**. Symbol size is proportional to number of genes. Uncorrected *p*-value < 10^−5^, from the right-sided hypergeometric test. White symbols were not enriched and were added to highlight the hierarchy between categories within Reactome structure.

To evaluate the susceptibility of both normal and reverse polarity organoids to SARS-CoV-2 we first conducted a pilot study with fetal and pediatric organoids. Normal polarity fetal and pediatric gastric organoids embedded in Matrigel were readily infected by SARS-CoV-2, as demonstrated by the presence of viral double-stranded RNA (dsRNA) in the cytosolic compartment of cells (Supplementary Fig. 4a), nevertheless titration of the culture supernatants by focus forming assay (FFA) failed to detect viable virus, at any time after infection. On the other hand, infection of RP-GOs was demonstrated by immunofluorescence. For this reason, a parallel comparison across fetal, pediatric, and adult age organoids was conducted using RP-GOs. After a 2-h infection, organoids were cultured up to 96 h in suspension and checked for structural integrity and viability by visual examination on a daily basis (Fig. 5a). Immunofluorescence staining of the coronavirus nucleocapsid protein (NP CoV) and dsRNA (Fig. 5b and Supplementary Fig. 4b) indicated the susceptibility to infection of all organoids irrespective of the donors’ age. Interestingly, NP CoV was detected in the same cells where cleaved caspase 3 (CCAS3) was observed, indicating that SARS-CoV-2 infected gastric cells undergo programmed cell death (apoptosis), as shown in Fig. 5c. We then analyzed the yield of infectious progeny virus and genome copies in culture supernatants collected at 0, 24, 48, and 72 h post-infection (Fig. 5d). Pediatric and late fetal RP-GOs recorded the highest infectious titers, with peak values of 10^4^ FFU/ml, at 72 h post-infection, significantly higher than early fetal and adult stage organoids, for whom the majority of values was either below or around the limit of detection of the FFA. To confirm the poor susceptibility of adult undifferentiated organoids, we repeated the experiment with the 45-years-old RP-GOs, using both the standard (MOI of 0.5) and a 10X challenge dose (MOI of 5). In this experiment, we observed temporary modest increases in genome copies, but we could not detect infectious viral progeny in the supernatant (Supplementary Fig. 5a).

### SARS-CoV-2 infection induces a moderate antiviral state in late fetal and pediatric organoids

Next, we performed RNA-seq analysis on non-infected and infected organoids samples. Interestingly, we identified very few genes significantly down-regulated while the majority of DEGs were up-regulated and could be seen only in samples from PCW 20 (30 DEGs) and pediatric organoids (32 DEGs) (Fig. 5e). Of these genes, 23 were common at these two developmental stages, such as *BST2, CMPK2, DDX58, DHX58, EIF2AK2, HERC5, HERC6, IFI44, IFI44L, IFIH1, IFIT1, IFIT2, IFIT3, IRF7, IRF9, ISG15, LAMP3, MX1, OAS2, PARP9, RSAD2, STAT1, TRIM22*. Given that apparent minimal susceptibility of adult organoids to SARS-CoV-2, we tested if a higher dose could elicit a stronger transcriptional response (Supplementary Fig. 5b). At these conditions only one gene was detected as significantly up-regulated after infection, *NLRP2*, and four genes were down-regulated (*ALDH3A1*, *CXCL8*, *IL1R2*, *KRT15*). We then focused on the analysis of the overall 39 genes that we found to be up-regulated in PCW 20 and pediatric organoids. More than 50% of them (20/39) were previously found to be DEGs in a literature survey of transcriptomic data on SARS-CoV, where 38 genes were identified as DEGs at the intersection of at least 9 studies (Fig. 5f). Among the common DEGs, some were first responders to the infection process, like the *DDX58* and *IFIH1* encoding the viral RNA sensors RIG-I and MDA5 respectively^31^, and their regulators, such as *DHX58*; others more downstream players of the response, such as *OAS2* that is activated by detection of dsRNA to inhibit viral replication, *IFIT2* that inhibits the expression of viral mRNAs, and BST2 that limits viral secretion. The overall transcriptional profile of the 39 DEGs identified in this study is shown in Fig. 5g, also for samples where these DEGs were not differentially expressed in response to the infection. *IFI44L* showed the highest fold change both in PCW 20 and in 11-year old samples. This gene was previously found to be a marker of viral infection compared to bacterial infection^32^ and more recently described as a negative modulator of innate immune responses induced after virus infections^33^. Type I, II, and III interferon (IFN) transcripts were not differentially expressed between non-infected and infected RP-GOs.

We then performed an enrichment analysis within the Reactome database to understand the functional implications of the DEGs up-regulation after the infection in PCW20 and 11-year samples (Fig. 5h). Interestingly, the majority of DEGs fell within pathways associated with the innate response to viral infection, particularly those involved in the regulation of type I IFN alpha/beta by cytoplasmic pattern-recognition receptors (PRRs) such as *RIG*-I and *MDA5*, and the expression of IFN stimulated genes (*ISGs*). Moreover, to capture more subtle differences between non-infected and infected samples that do not emerge in the DEG analysis, we performed a Quantitative Set Analysis for Gene Expression (QuSAGE)^34^ within the Gene Ontology database (Supplementary Data 3). Categories related to the IFN response to the infection were identified in three of the sample groups (PCW11, PCW20, and pediatric). Other categories were reported for completeness but require further studies to understand their relevance.

### Adult origin RP-GOs differentiation promotes SARS-CoV-2 viral progeny release

**Fig. 6:**
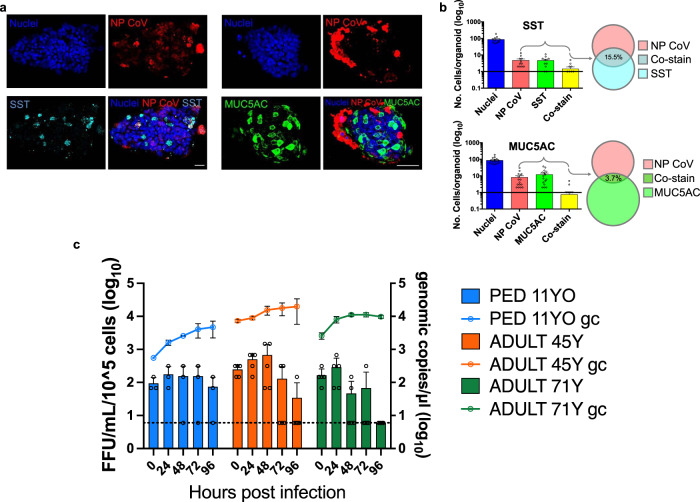
SARS-CoV-2 infection of human pediatric and adult gastric organoids. **a** Immunofluorescence panels for undifferentiated pediatric RP-GOs, showing NP CoV in red and somatostatin (SST) in cyan (left panel), and NP CoV in red and mucin 5AC (MUC5AC) in green (right panel). Scale bars 100 µm. **b** Cell counting of infected cells, SST, MUC5AC positive and co-positive cells per organoid of pediatric origin. Mean ± SEM (SST *n* = 11 organoids; MUC5AC *n* = 16 organoids). **c** Graphs of SARS-CoV-2 replication in pediatric and adult differentiated RP-GOs. Culture supernatants collected at 0, 24, 48, 72, and 96 h after infection with SARS-CoV-2 (MOI of 10) were titrated for the presence of live virus and viral genome copies by the FFA and qRRT-PCR, respectively. The dotted lines indicate the lower limit of detection. Black circles indicate single data points. Mean ± SD (*n* = 5 experimental replicates).

We then investigated the tropism for the different gastric cell sub-types in pediatric RP-GOs, to evaluate which cells were more susceptible to viral infection. We were able to assess that 15.5% of somatostatin-secreting endocrine delta-cells were infected, while only 3.7% of mucin 5AC-secreting mucous cells were infected by the virus (Fig. 6a, b).

To understand whether the modest replicative fitness of SARS-CoV-2 in adult organoids depended on the stage of differentiation, we differentiated both pediatric and adult organoids, by selective withdrawal of WNT^19^. These lines were then subsequently infected with a 20X dose (MOI of 10). In this case we observed a clear replication of the virus, as demonstrated by the increase in virus RNA concentrations over time and the presence of viral infectious progeny in the supernatant, over several days (Fig. 6e). Interestingly, infectious titers did not statistically differ between ages.

## Discussion

Reliable in vitro models capable of reproducing complex in vivo systems are becoming increasingly important in life sciences and play a crucial role in the investigation of emerging pathogens like SARS-CoV-2. In the context of the COVID-19 pandemic, it is still unclear how gastrointestinal virus replication might affect the clinical outcome of infection, the development of immunity and the transmission dynamics in the population. While it has been shown that SARS-CoV-2 is frequently detected in  fecal samples of affected children and adults^5,6,35^, it remains to be determined if the virus is able to produce a primary infection throughout the entire GI tract, or if its presence could be related in part to a passive transport of contaminated sputum coming from the upper respiratory tract. Most importantly, the ability of SARS-CoV-2 to persist in the GI tract after respiratory clearance, has not yet been fully elucidated in terms of viral infectivity, possibly impairing important public health and policy measures for the control of the disease. These concerns are particularly relevant in children who appear on average to suffer a less severe respiratory illness compared to adults, despite recording more prominent GI symptoms, hyperinflammatory shock syndrome (Paediatric Multisystem Inflammatory Syndrome—Temporally Associated with SARS-CoV-2, PIMS-TS)^36^, or acting as relatively asymptomatic carriers of the virus.

Two important studies show that the main SARS-CoV-2 receptor ACE2 is highly expressed on differentiated enterocytes and that intestinal organoids derived from the small intestine can be easily infected by SARS-CoV-2^15,16^. Interestingly, intestinal organoids derived from both human and horseshoe bats are fully susceptible to SARS-CoV-2 infection and sustain robust viral replication. Although vertical transmission of SARS-CoV-2 seems to be anecdotal, it is still unclear if this lack of infection relates to the inability of the virus to migrate through the placenta^37^, to the low susceptibility of the fetal cells to infection, or simply on low viral load. Whereas human fetal intestinal organoids have already been reported^21^, a reliable 3D culture in vitro model of human gastric mucosa at different developmental stages has been challenging to achieve.

In this study, we describe successful derivation of human stomach organoids across developmental stages, and we demonstrate that gastric cells are susceptible to SARS-CoV-2 infection. Furthermore, we describe how a reverse polarity organoid model can help expose the apical domains to direct contact with the surrounding microenvironment, so that pathogens can easily access surface receptors on the cells.

To overcome inter-operator and inter-laboratory variability, resulting in the generation of less-reproducible data, we decided to prevent the system perturbation and infect human gastric organoids under steady-state conditions. Taking advantage of a polarity reversion study^29^, we generated cultures of RP-GOs in suspension of fetal, pediatric, and adult origin.

In this condition of exposed apical polarity and absence of surrounding Matrigel, we could infect organoids and readily titrate the infectivity of the progeny virus, recording infection level comparable to those shown by Zhou et al.^16^. Interestingly, when we infected pediatric gastric organoids through shearing and re-embedding in Matrigel, infection was achieved but failed to detect virus in the supernatants, indicating this approach as suboptimal for our purposes, although we do not exclude that further optimization of the shearing technique in our laboratory could have led to better results.

We demonstrated that the RP-GOs are fully susceptible to SARS-CoV-2 infection and observed that late fetal and pediatric organoids allow higher replication of the virus, while early fetal and adult organoids, although infected, released significantly lower amounts of infectious viral progeny when cultured in expansion medium. Quantification of gene transcripts coding for the viral receptors ACE2 and TMPRSS2 suggested that the observed levels of replication could depend on differences in the density of ACE2 receptors, given that few counts per million were recorded for this gene in the adult organoids. Although we could not run the same analysis on adult stomach biopsies, our data on ACE2 expression and susceptibility to infection are in keeping with the literature indicating that adult gastric cells express lower levels of ACE2 compared to intestinal cells^30^, but also that these cells have lower co-expression of ACE2 and TMPRSS2 at single-cell level^38^. Nonetheless, variation in the protein-to-mRNA ratio across organoids of different developmental stage should be taken into account and receptivity investigated in future work. Among the gastric sub-types analyzed, we found that 15.5% of somatostatin-positive cells were infected by SARS-CoV-2, while only 3.7% of mucin 5AC-positive cells were infected. We can therefore conclude that somatostatin-secreting Delta cells are one of the susceptible cell types to infection, with no information available on other cell types, given their relative low abundance in this RP-GO model. Interestingly, the differentiation of pediatric and adult organoids coupled with a high MOI of 10 led to the infection of both pediatric and adult organoids and a similar replication kinetic. Further investigation is required to explain whether differentiation and the presence of mature cell types increased the susceptibility of adult organoids to infection, or if this was attributable to the higher challenge dose.

In RP-GOs, immunofluorescence staining for the nucleocapsid indicated a clear cytosolic localization of this protein that in some cells was associated with the presence of the cleaved caspase 3, confirming the occurrence of apoptosis in the gastric compartment^15^. Apoptosis in infected GI mucosal cells might account at least in part for the frequent abdominal pain, vomiting, and diarrhea described in COVID-19 patients^39^, in particular in pediatric populations. Apoptosis is one of the key mechanisms of cells to restrict viral infections by destruction of the cellular machinery indispensable for virus replication. On the other hand, selected viruses have evolved diverse adaptative strategies to control this phenomenon in their favor^40^. To this respect, SARS-CoV was shown to replicate in vitro to high titers in cells undergoing apoptosis and to low titers in cells where cytopathic effect was limited and a persistent infection was established^41^. Interestingly, induction of apoptosis for SARS-CoV was proved to be caused by a nuclear localization of the nucleocapsid protein that in turn resulted in its cleavage by caspases 6 and 3. The precise mechanism underpinning nucleocapsid cleaving, apoptosis and the replication efficiency of SARS-CoV-2 remains unexplored. We believe that similar mechanistic studies are of great interest to decipher the pathology of SARS-CoV-2 in the GI system and its implications on virus shedding and transmissibility.

RP-GOs infected with SARS-CoV-2 shared a transcriptional footprint surprisingly similar to those described for infected human small intestine enteroids^15,16^ in which type I IFN genes were either poorly expressed or undetectable, despite enterocytes and gastric cells displaying moderate levels of ISGs primarily involved in the recognition of viral RNA. Moreover, our transcriptional data are in considerable agreement with clinical and experimental profiles derived from COVID-19 patients, infected normal human bronchial epithelial cells and in vivo studies in ferrets^42^ that highlighted a negligible expression of genes of the IFN family but a robust expression of chemokines and ISGs. Our data provide evidence in support of the hypothesis that pathogenesis of COVID-19 is at least in part dependent on a reduced innate antiviral response and an unbalanced cytokine production. Nevertheless, in our model chemokines were not differentially expressed, whereas in small intestine organoids, Zhou et al.^16^ reported DEGs coding both chemokine receptors and ligands. Since we conducted a bulk RNA-seq analysis and the number of infected cells in our organoids were still a minority, we speculate that many processes specific to infected cells, most likely did not reach a statistically significant level and might have gone undetected, hence imposing a cautionary approach in our interpretation of the data. Interestingly, a large overlap of DEGs with previous transcriptomic studies of SARS-CoV infection was found, including the peculiar feature of a limited/absent type I IFN induction and the recruitment of a subset of cytoplasmic PRRs. Similar to SARS-CoV, in which ORF3B and ORF6 are the main antagonists of IFN, a study by Konno et al.^43^ indicates SARS-CoV-2 ORF3B protein as a potent IFN inhibitor, supporting ours and the published transcriptomic data herein discussed.

Our gastric organoid system offers a unique tool to characterize the replication of viruses and some of the associated pathological consequences of infection. This innovative model could represent an in vitro scalable platform for the development and testing of antiviral drug candidates targeting the GI system. A deeper understanding of the pathogenic mechanism underpinning the viral colonization of the GI system will potentially expand the available therapeutic options for the inhibition and preventing of GI infection, in an attempt to suppress viral shedding and halt spreading of the disease. The clinical importance of our findings relates to the worrisome phenomenon of prolonged shedding of SARS-CoV-2 from the GI tract and calls for further research to assess the risk of vertical transmission in infected women. Defining ages of susceptibility and identifying target anatomical sites will prove of crucial importance for the implementation of sensitive and sustainable diagnostic screening for the identification of contagious asymptomatic patients.

## Methods

### Isolation of human gastric stem cells

Human fetal stomachs were dissected from tissue obtained immediately after termination of pregnancy from 8 to 21 PCW (post conception week), in compliance with the bioethics legislation in the UK. Fetal samples were sourced following patient informed consent via the Joint MRC/Wellcome Trust Human Developmental Biology Resource with Research Tissue Bank ethical approval (08/H0712/34+5). Human pediatric gastric surgical biopsies were collected after informed consent, in compliance with all relevant ethical regulations for work with human participants, following the guidelines of the licenses 08ND13 and 18DS02 (NHS Health Research Authority—East of England—Cambridge Central Research Ethics Committee). Adult organoids were obtained through material transfer agreement and were previously derived in the Hubrecht Institute under a study approved by the ethical committee of the University Medical Center Utrecht (UMCU; the Netherlands) and in accordance with the Declaration of Helsinki. Patients gave informed consent in compliance to Dutch law and all relevant ethical regulations regarding research involving human participants^25^. We derived a biobank of 5 early fetal lines (from CS23 to PCW 11), 6 late fetal lines (from PCW 18 to PCW 21), 4 pediatric lines (from 4 months to 11 years-old), and 2 adult lines (45 and 71 years-old).

Stomach biopsies were collected in ice-cold sterile phosphate buffered solution (PBS—Sigma-Aldrich) and processed within a few hours of collection. Gastric gland stem cells were isolated from specimens following a well-established dissociation protocol^19,44^. Briefly, fetal stomachs were cut open longitudinally along the lesser curvature, while ≅0.5 cm^2^ pediatric biopsies where processed as they were obtained. Specimens were cold-washed in a plate with chelating buffer (sterile Milli-Q water (Merck Millipore) with 5.6 mmol/L Na_2_HPO_4_, 8.0 mmol/L KH_2_PO_4_, 96.2 mmol/L NaCl, 1.6 mmol/L KCl, 43.4 mmol/L sucrose, 54.9 mmol/L d-sorbitol, 0.5 mmol/L dl-dithiothreitol, pH 7, all from Sigma-Aldrich). Mucus was removed with a glass coverslip and mucosa was stripped from muscle layer. Tissue was cut in small pieces, transferred in a 15 mL tube in new chelating buffer and pipetted repeatedly. Supernatant was discarded and 10 mL of 10 mM EDTA was added and incubated for 10 min at room temperature. EDTA was discarded and mucosa pieces were washed in ice-cold PBS with Ca^2+^/Mg^2+^ (Sigma-Aldrich). Tissue was transferred to a new 10 cm plate on ice and pressure was applied on top with a sterile 3.5 cm plate, to release the glands from the mucosa. Glands were collected in ice-cold ADMEM + + + , composed of Advanced DMEM F-12, 10 mM HEPES, 2 mM Glutamax, 1% Pen/Strep (all from Thermo Fisher Scientific). Medium containing the glands was filtered through a 40 μm strainer and centrifuged at 300 g for 5 min at 4 °C. Supernatant was discarded and ice-cold liquid Matrigel^®^ Basement Membrane Matrix Growth Factor Reduced (GFR) (Corning 354230) was added to the pellet and thoroughly resuspended. Droplets of 30 μL were aliquoted on warmed multi-well plates and incubated for 20 min at 37 °C for gelation. Medium was added and changed every 3 days. For medium recipes refer to Supplementary Table 2.

### Passage of organoids

Cell were passaged every 6–8 days. To passage the organoids, Matrigel droplets were disrupted by pipetting in the well and transferred to tubes on ice. Cells were washed with 10 mL of cold basal ADMEM+++ and centrifuged at 200×*g* at 4 °C. (First method) For single-cell dissociation, supernatant was discarded, and the pellet resuspended in 1 mL of TrypLE (Thermo Fisher) and incubated for 5 min. After incubation organoids were disaggregated by pipetting, and 10 mL of ice-cold ADMEM+++ was added to dilute and inhibit TrypLE. (Second method) For standard organoid passage during expansion, the organoid pellet was resuspended in 1.5 mL of ice-cold ADMEM+++ and organoids were manually disrupted by narrowed (flamed) glass pipette pre-coated in BSA 1% in PBS, to avoid adhesion to the glass. Cells were washed, pelleted, and supernatant discarded. Almost-dry pellets of disaggregated organoids (or single cells) were thoroughly resuspended in cold liquid Matrigel, aliquoted in 30 µL droplets in pre-warmed multi-well plates and incubated at 37 °C for 20 min to form a gel. Rho-kinase inhibitor (Tocris) was added to single-cell dissociated organoids. Medium was added and changed every 3 days.

### Polarity reversion and organoid differentiation

Fully grown gastric organoids at day 7 after single-cell disaggregation were removed from surrounding extracellular matrix using a modified published protocol^29^. Matrigel was dissolved with 60 min treatment of the droplets with Cell Recovery Solution (Corning) at 4 °C. Whole organoids were retrieved from the plates using 1% BSA-coated cut-end tips and transferred to 1% BSA-coated 15 mL tubes. Cells were extensively washed with ice-cold PBS and centrifuged at 200 g for 5 min at 4 °C. Supernatant was discarded, the pellet was resuspended in complete medium and transferred to non-tissue culture treated low-adhesive multiwell plates (pre-coated in 1% BSA). Organoids were cultured in suspension for 3 days to allow reversion of polarity, before use in infection experiments.

For gastric organoid differentiation, we cultured the organoids in Matrigel in expansion medium for 10 days after single-cell disaggregation, followed by 4 days of polarity reversion in differentiation medium^19^ (withdrawal of Wnt-3A and CHIR 99021). The RP-GOs differentiated were then infected in differentiation medium.

### DNA isolation and single-nucleotide polymorphism analysis

Full grown 7-days-old organoids from passages 11–16 were washed from Matrigel with ice-cold PBS and DNA was extracted using DNeasy Blood & Tissue Kit (Qiagen), following manufacturer’s instructions. DNA in water was analyzed using 8 μL volume at 75 ng/μl concentration per sample. SNP array Infinium Core-24 v1.1 Kit (Illumina) was used according to manufacturer’s instructions. Results were analyzed through GenomeStudio software (Illumina). We exported representative images of the chromosome viewer/B allele frequency, for each analyzed sample.

### RNA isolation

For RNA isolation from stomach tissues, (i) pediatric stomach biopsies consisted of only mucosal layer from surgical samples; (ii) Late fetal stomachs were cut open and mucosal layer was isolated; and (iii) early fetal stomachs were processed with no layer isolation, given the small size of the samples. Mucus was removed from all the samples with a glass coverslip to prevent RNA loss during the isolation protocol, and tissues washed in ice-cold PBS. Then the tissues were finely cut with a scalpel on a Petri-dish on ice and transferred to 1.5 mL tubes.

RNA was isolated from cultured organoids in Matrigel with 30 min treatment of the droplets with Cell Recovery Solution (Corning) at 4 °C. Cells were then washed in ice-cold PBS to remove matrix leftovers that could interfere with RNA isolation. Organoids were centrifuged at 200×*g* at 4 °C and supernatant discarded.

Dry pellets of tissues and organoids were lysed with RLT buffer (Qiagen). RNA was isolated with RNeasy Mini Kit (Qiagen) following manufacturer’s instructions. Total RNA was quantified using the Qubit 2.0 fluorimetric Assay (Thermo Fisher Scientific).

### Real-time PCR

RNA reverse transcription was performed using the High-Capacity cDNA Reverse Transcription Kit (Thermo Fisher), according to the manufacturer’s instructions. Reverse transcription was done using the T100 thermal cycler (Bio-Rad). The qRT-PCR was performed with TaqMan gene expression assay probes (Thermo Fisher) according to the manufacturer’s instructions. The following probes (all from Thermo Fisher) were used: GAPDH (glyceraldehyde 3-phosphate dehydrogenase), LGR5 (leucine-rich repeat-containing G-protein coupled receptor 5), AXIN2 (axin-like protein), MUC5AC (mucin 5AC), MUC6 (mucin 6), PGA5 (pepsinogen A5), SST (somatostatin), GAST (gastrin), CHGA (chromogranin A), and ATP4B (ATPase H + /K + transporting subunit beta). Reactions were performed on Step One Plus Real-Time PCR System (Applied Biosystems) and results were analyzed with StepOne (Version 2.3) software (Life Technologies). GAPDH expression was used to normalize Ct values for gene expression, and data were shown as relative fold change to controls (early fetal stage), using ∆∆Ct method, and presented as mean ± SEM, with *p*-value *<0.05, **<0.01, ***<0.001.

### RNA Seq and transcriptome bioinformatic analyses

For RNA-seq data of original tissues and organoids with standard polarity, total RNA (100 ng) from each sample was prepared using QuantSeq 3′ mRNA-Seq Library prep kit (Lexogen GmbH) according to manufacturer’s instructions. The amplified fragmented cDNA of 300 bp in size were sequenced in single-end mode using the Nova Seq 6000 (Illumina) with a read length of 100 bp. Illumina novaSeq base call (BCL) files were converted into fastq files through bcl2fastq (version v2.20.0.422) following software guide. Sequence reads were trimmed using bbduk software (bbmap suite 37.31), following software guide, to remove adapter sequences, poly-A tails and low-quality end bases (regions with average quality below 6). Alignment was performed with STAR 2.6.0a^45^ on hg38 reference assembly obtained from cellRanger website (Ensembl 93), following online site guide. The expression levels of genes were determined with htseq-count 0.9.1 by using cellRanger pre-build genes annotations (Ensembl Assembly 93). All transcripts having <1 CPM in <4 samples and percentage of multimap alignment reads > 20% simultaneously were filtered out.

For RNA-seq data of non-infected and infected RP-GOs, a total of 600 pg of RNA was used as input for the synthesis of cDNA with the SMART-Seq v4 Ultra Low Input RNA Kit for Sequencing (Takara Bio USA, Mountain View, CA, USA). Manufacturer suggested protocol was followed, with minor modifications. 75 pg of DNA generated with SMART-Seq v4 Kit were used for preparation of library with NEXTERA XT DNA Library Preparation kit (Illumina Inc., San Diego, CA, USA), following suggested protocol. Libraries were sequenced in pair-end mode using a Nova Seq 6000 sequencing system on an SP, 100 cycles flow cell (Illumina Inc., San Diego, CA, USA). Illumina novaSeq base call (BCL) files were converted into fastq files through bcl2fastq (version v2.20.0.422) following software guide. Alignment was performed with STAR 2.6.0a^45^ on hg38 reference assembly obtained from the Gencode website (primary assembly v. 32). Transcripts estimated counts were determined with RSEM 1.3.0^46^ by using the Gencode v.32 genes annotations. All genes having <1 CPM in less than 2 replicates of the same condition were filtered out.

Differentially expressed genes (DEGs) were computed in R (version 3.5) with edgeR (version 3.24)^47^, using a mixed criterion based on p-value, after false discovery rate (FDR) correction by Benjamini-Hochberg method, <0.05 and absolute log_2_(fold change) higher than log_2_(1.5). This analysis was paired between non-infected and infected samples derived from the same original sample. For RNA-seq data of organoids with spontaneous polarity, DEGs were clustered according to a flat, increasing, or decreasing profile according to the differential expression analysis between pairs of time points. Principal Component Analysis was performed by Singular Value Decomposition (SVD) on log_2_(CPM + 1) data, after centering, using MATLAB R2017a (The MathWorks). DEGs over-representation analysis of Gene Ontology (GO, https://www.ebi.ac.uk/GOA) and Reactome (https://reactome.org/) categories was performed using ClueGO (version 2.5.4)^48^. Reactome hierarchy was visualized using ClueGO within Cytoscape (version 3.5)^49^. Hierarchical clustering of DEGs was performed on median-centered log_2_(CPM + 1) data in MATLAB, using Euclidean distance and complete linkage. Log-normalized expression data were analyzed by the Quantitative Set Analysis for Gene Expression (QuSAGE, version 2.16)^34^ Bioconductor (version 3.8) package.

### Virus isolation and cell lines

Vero E6 cells (ATCC® CRL 1586™) were maintained in Dulbecco’s modified Eagle’s medium (DMEM, Thermo Fisher) supplemented with 10% fetal calf serum (FCS), penicillin (100 U/ml), and streptomycin (100U/ml) (all from Thermo Fisher) at 37 °C in a humidified 5% CO_2_ incubator. The SARS-CoV-2 isolate was obtained from a nasopharyngeal swab collected from a 14-year-old boy during routine diagnostic activities conducted at the University Hospital of Padua (Italy). Legally authorized representatives of the subject provided written consent for the collection and use of biological specimens for research purposes (Protocol N° 0070714; amendment number 71779; Ethics Committee of the Azienda Ospedale Università di Padova). Briefly, the swab viral transport medium was filtered through a 0.22 µm filter, serially diluted and incubated onto a confluent layer of Vero E6 cells, for 5 days. To ensure purity of the viral isolate, the supernatant of the highest dilution in which cytopathic effect was visible was tested for the presence of 21 human respiratory pathogens including SARS-CoV-2, using the QIAstat-Dx Respiratory SARS-CoV-2 Panel (Qiagen). Viral stocks were produced infecting at a multiplicity of infection (MOI) of 1 Vero E6 cells cultured in DMEM supplemented with 2% FCS, penicillin (100 U/ml) and streptomycin (100 U/ml) and incubating the cells for 48 h. Supernatants were collected when 80% cells exhibited cytopathic effect and cleared by low- speed centrifugation before being stored at −80 °C. Reverse polarity gastric organoids derived at PCW11, PCW 20, pediatric and adult stages, and normal-polarity organoids were infected by trained virologists in a biosafety level 3 (BSL3) laboratory. All infections in this paper were performed using the third culture passage of the original isolate.

### Infection of organoids in suspension

Intact organoids were embedded in two 3 µl droplets of Matrigel per well, in 24-well plates. Embedded organoids were washed once in DMEM and infected at a MOI of 0.5 by incubation with 250 µl of an expansion medium viral suspension for 2 h. After removal of the inoculum, organoids were washed twice with a DMEM solution and 400 µl of complete medium were added to each well to maintain the culture at 37 °C with 5% CO2. Differentiated and undifferentiated RP-GOs were infected at MOI of 0.5, 5, and 10 by incubation with 250 µl of an expansion medium viral suspension for 2 h. After infection, organoids were washed twice in DMEM to remove unbound virus. RP-GOs were dispersed in a 400 µl expansion medium at 37 °C with 5% CO2. For all organoid cultures 50 µl of supernatant were harvested at 0, 24, 48, and 72 h post infection. An equal volume of expansion medium replaced the sampled supernatant at each collection time. An extra sample at 96 h post infection was collected for the RP-GOs. Samples were stored at −80 °C before titration through the FFA.

### Virus titration by focus forming assay

Supernatants of organoid cultures and aliquots of viral stocks were serially diluted and incubated on confluent monolayers of Vero E6 cells, in 96-well plates, for 1 h. Culture medium formulation was the same used for virus propagation. After infection, the inoculum was removed and an overlay of MEM, 2% FBS, penicillin (100 U/ml) and streptomycin (100 U/ml), and 0.8% carboxy methyl cellulose was added. After 27 h, the overlay medium was removed and cells were fixed in a 4% paraformaldehyde (PFA) in phosphate buffered solution (PBS), for 30 min at 4 °C. Upon removal, cells were permeabilized by incubation with a 0.5% Triton X-100 solution for 10 min. Immunostaining of infected cells was performed by incubation of the J2 anti-dsRNA monoclonal antibody (1:10,000; Scicons) for 1 h, followed by 1-h incubation with peroxidase-labeled goat anti-mouse antibodies (1:1000; DAKO) and a 7 min incubation with the True Blue™ (KPL) peroxidase substrate. Solution of 1% bovine serum albumin and 0.05% Tween-80 in PBS was used for the preparation of working dilutions of immuno-reagents. After each antibody incubation, cells were washed 4 times through a 5 min incubation with a 0.05% Tween-80 PBS solution. Focus forming units (FFU) were counted after acquisition of pictures at a high resolution of 4800 × 9400 dpi, on a flatbed scanner.

### Staining and immunofluorescence

Human gastric tissues were fixed in 4% paraformaldehyde (PFA—Sigma-Aldrich) for 2 h and embedded in paraffin wax, then cut at 7 μm on a microtome. Hematoxylin and Eosin (H&E) tissue slides were stained according to manufacturer’s instructions with Hematoxylin and Eosin (H&E) (Thermo Fisher).

Cultured organoids were removed from Matrigel with 30 min treatment of the droplets with Cell Recovery Solution at 4 °C. RP-GOs were fixed in suspension by transferring them to 1% BSA pre-coated 1.5 mL tubes, centrifuging and resuspension in 4% PFA for 20 min in rotation. PFA was discarded and quenched with 0.1 M NH_4_Cl for 1 h in rotation. Matrigel droplets (3 μL on glass slides) with embedded organoids were fixed in 2% PFA for 20 min at RT, and then washed.

Immunostaining was performed by blocking and permeabilizing the tissue slides with PBS + Triton X-100 0.1% with BSA 0.5%. Organoid whole-mounts were blocked and permeabilized with PBS + Triton X-100 0.5% with BSA 1% for 2 h at room temperature in rotation. Primary antibodies were incubated in blocking buffer for 24 h at 4 °C in rotation and extensively washed in PBS + Triton X-100 0.1%. Secondary antibodies were incubated overnight at 4 °C in rotation and extensively washed. Slides were mounted in mounting medium, while floating organoids were moved to a glass-bottomed Petri dish and blocked with a coverslip on top. The full list of primary and secondary antibodies is presented in Supplementary Table 3.

### Image acquisition

Organoids were imaged in bright field using a Zeiss Axio Observer A1. Immunofluorescence images of whole-mount staining and sections were acquired on a confocal microscope Zeiss LSM 710. Infected organoid immunofluorescence images were acquired on a Leica TCS SP5.

### Statistics and reproducibility

Statistical analyses were performed using the following software: MATLAB (v. R2017a) for PCA, pie plot, bar plot, hierarchical clustering with proteomic and RNA-seq data. GraphPad Prism Mac (v. 6.0 h) was used with all other graphs and charts. Numerosity of representative experiments: Fig. 1c–e representative of *n* = 3. Figure 2a, b representative of *n* = 3. Figure 2c and Suppl. Fig. 3a representative of *n* = 50 stained organoids per line. Figure 4b, c representative of six-well plates full of organoids, with each polarity reversion experiment repeated *n* = 8 for each cell line. Figure 4f representative of *n* = 3 tissue slides per sample. Figure 4g representative of *n* = 50 stained organoids per line. Figure 5a representative of *n* > 100 organoids per line. Figure 5b, c and Suppl. Fig. 4a, b representative of *n* = 50 stained organoids per line. Figure 6a representative of *n* = 20 stained organoids per line. All other numerosity and statistical tests used are reported in the figure legends.

### Reporting summary

Further information on research design is available in the Nature Research Reporting Summary linked to this article.

## Supplementary information

Supplementary Information

Reporting Summary

Description of Additional Supplementary Files

Supplementary Data 1

Supplementary Data 2

Supplementary Data 3

## Data Availability

The authors declare that all data supporting the findings of this study are available within the article, its Supplementary Information, Source Data files, and online deposited data. The Gastric QuantSeq RNA-seq data generated in this study, presented in Figs. 3a–f and 4d, and Supplementary Fig. 2a–g have been deposited in the NCBI GEO database under accession code GSE153698. The Gastric SMART RNA-seq data presented in Figs. 4e and 5e–h, and Supplementary Figs. 3c and 5b have been deposited in the NCBI GEO database under accession code GSE153684. The Gastric SMART RNA-seq data presented in Fig. 4e, and Supplementary Fig. 5b have been deposited in the NCBI GEO database under accession code GSE184390. Source data are provided with this paper.

## Source data

Source Data
