# Supplementary material for: SARS-CoV-2 infection and replication in human gastric organoids
Source: Nat Commun. 2021 Nov 16;12:6610. doi: 10.1038/s41467-021-26762-2 (PMC8595698; doi:10.1038/s41467-021-26762-2)
Supplement: Supplementary file 1 — Supplementary Information [file 41467_2021_26762_MOESM1_ESM.pdf]

Supplementary Figures and Tables

SARS-CoV-2 infection and replication in human gastric organoids

Giovanni Giuseppe Giobbe[1,&,*], Francesco Bonfante[2,&], Brendan C. Jones[1], Onelia Gagliano[3], Camilla Luni[4], Elisa Zambaiti[1,3,5], Silvia Perin[1], Cecilia Laterza[3], Georg Busslinger[6], Hannah Stuart[3], Matteo Pagliari[2], Alessio Bortolami[2], Eva Mazzetto[2], Anna Manfredi[7,8], Chiara Colantuono[7,8], Lucio Di Filippo[7,8], Alessandro Pellegata[1], Valentina Panzarin[2], Nikhil Thapar[9], Vivian Sze Wing Li[10], Simon Eaton[1], Davide Cacchiarelli[7,11], Hans Clevers[6,12], Nicola Elvassore[1,3,4,13,*], Paolo De Coppi[1,14,*].

[1] Stem Cell and Regenerative Medicine Section, GOS Institute of Child Health, University College London, London, UK
[2] Lab. of Experimental Animal Models, Division of Comparative Biomedical Sciences, Istituto Zooprofilattico Sperimentale delle Venezie, Legnaro, Italy
[3] Veneto Institute of Molecular Medicine (VIMM), Padova, Italy
[4] Shanghai Institute for Advanced Immunochemical Studies (SIAIS), ShanghaiTech University, Shanghai, China
[5] Dept. Women's and Children's Health, University of Padova, Padova Italy
[6] Oncode Institute, Hubrecht Institute, Royal Netherlands Academy of Arts and Sciences (KNAW) and University Medical Center (UMC) Utrecht, Utrecht, Netherlands
[7] Telethon Institute of Genetics and Medicine (TIGEM), Armenise/Harvard Laboratory of Integrative Genomics, Pozzuoli, Italy
[8] Next Generation Diagnostic srl, Pozzuoli, Italy
[9] Gastroenterology, Hepatology and Liver Transplant, Queensland Children's Hospital, Brisbane, Australia
[10] Stem Cell and Cancer Biology Lab, the Francis Crick Institute, London, UK
[11] Department of Translational Medicine, University of Naples Federico II, Naples, Italy
[12] Princess Máxima Center (PMC) for Pediatric Oncology, Utrecht, Netherlands
[13] Dept. of Industrial Engineering, University of Padova, Padova, Italy
[14] Dept. of Specialist Neonatal and Paediatric Surgery, Great Ormond Street Hospital, London, UK
[&] These authors contributed equally: Giovanni Giuseppe Giobbe & Francesco Bonfante
* These authors jointly supervised this work: Giovanni Giuseppe Giobbe g.giobbe@ucl.ac.uk, Nicola Elvassore n.elvassore@ucl.ac.uk & Paolo De Coppi p.decoppi@ucl.ac.uk

Supplementary Figures

a

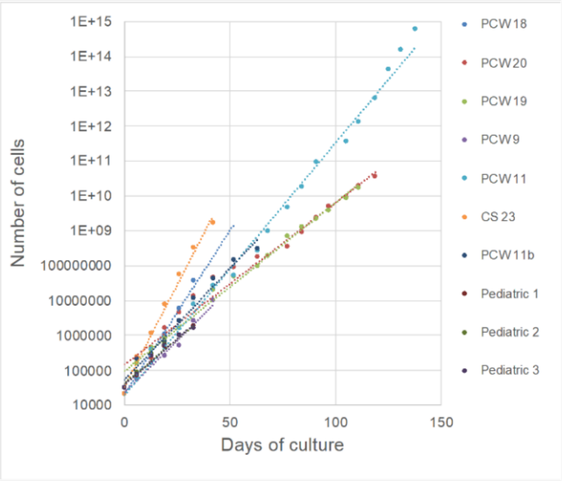

b

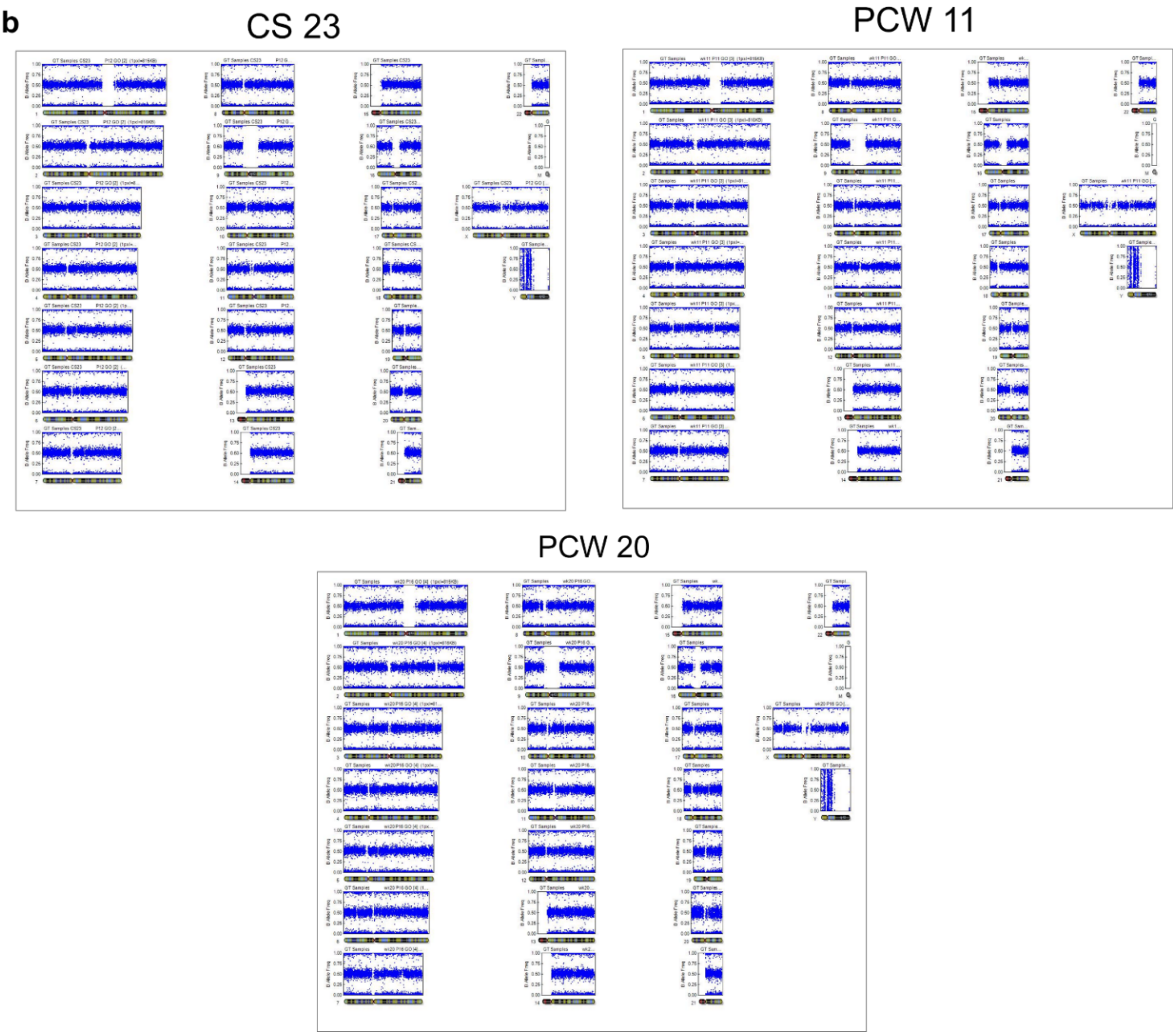

Supplementary Figure 1. a) Cumulative cell counts of single organoid lines during days of culture, up to a period of 5 months. b) Single nucleotide polymorphism (SNP) arrays. Representative image of the chromosome viewer and allele frequency presented for each analyzed fetal sample.

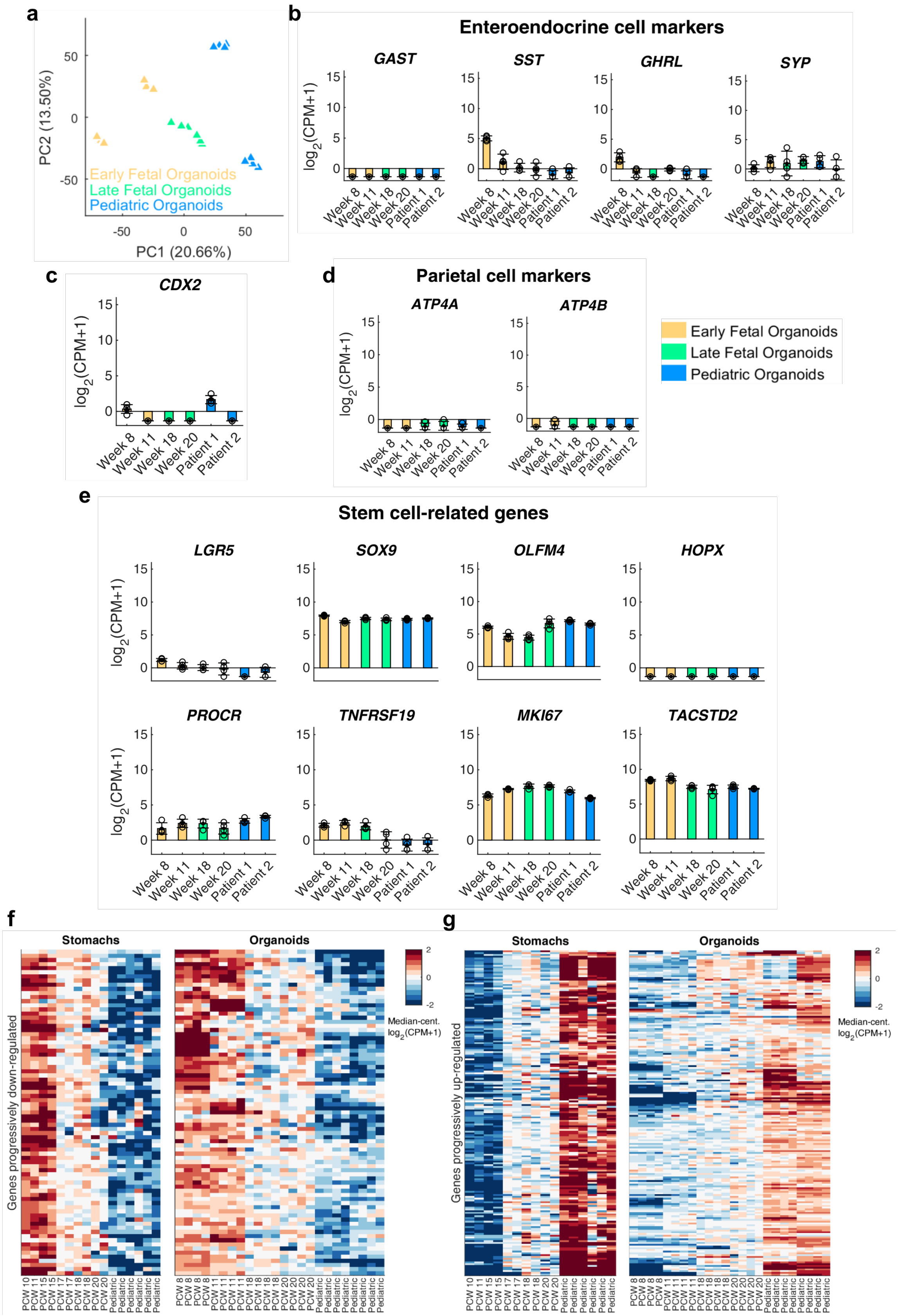

Supplementary Figure 2. Transcriptomic characterization of organoids from different stages of development. a) Principal Component Analysis (PCA) of RNA-seq organoid samples at different stages of development as indicated. Stages of tissue of origin for organoid derivation: early fetal (week 8-15), late fetal (week 17-20) and pediatric. b-e) Expression of selected genes in organoid samples: (b) enteroendocrine cell markers, (b) intestinal marker CDX2, (d) parietal cell markers typical of the corpus compartment, (e) putative genes identifying gastric stem cells. Red dots: single data points. Black error bar: Mean± SD (n = 4). CPM: count per million. f-g) Hierarchical clustering of genes progressively down- (f) and up- (g) regulated in samples derived from donors of increasing fetal and pediatric age, with similar trend in tissues and organoids. Gene expression is defined in terms of median-centered CPM. The full list of genes is reported in Supplementary Data 1.

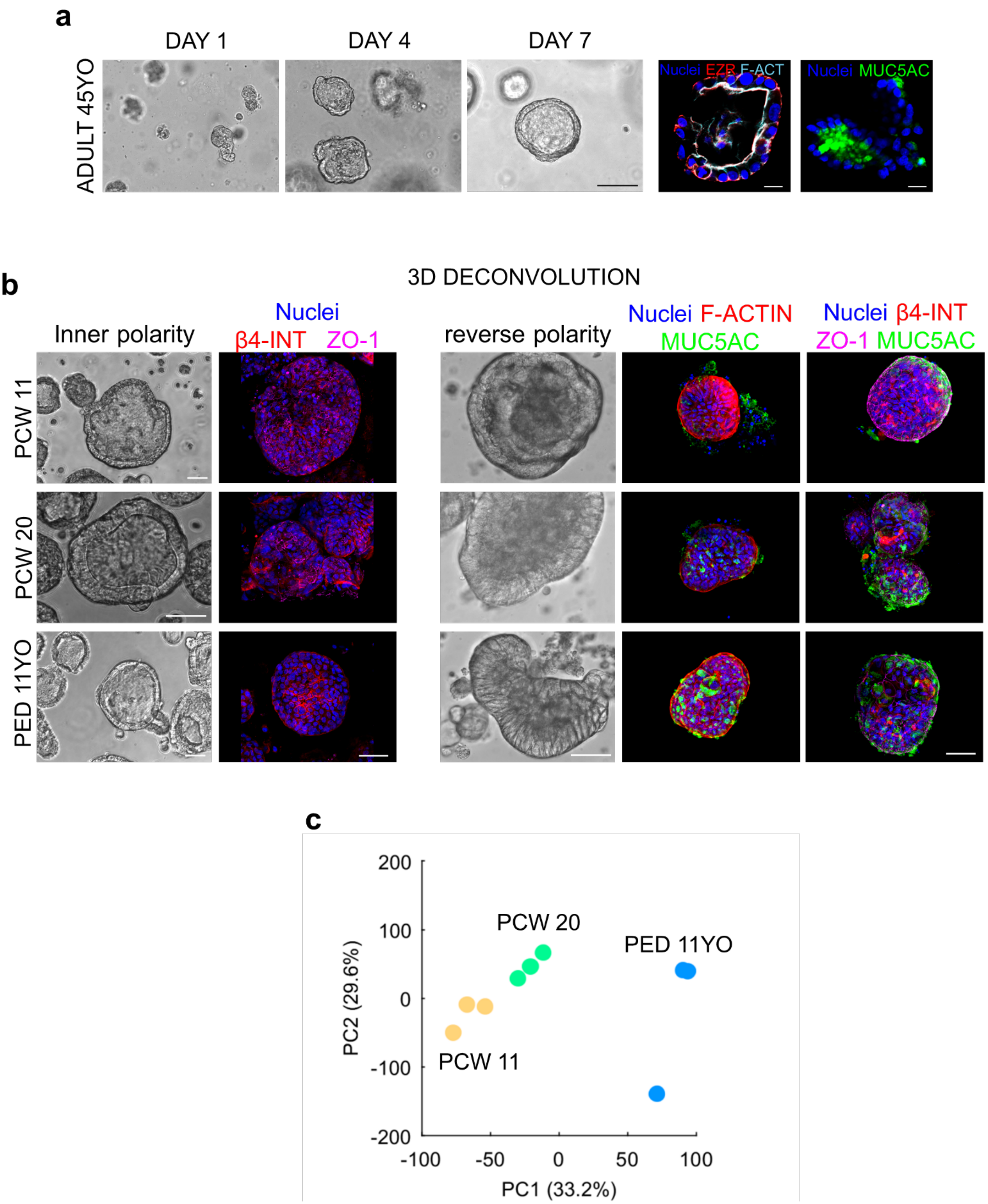

Supplementary Figure 3. a) Immunofluorescence panel for adult 45 years-old patient organoids showing ezrin (EZR) in red, f-actin (F-ACT) in cyan, mucin 5AC (MUC5AC) in green and nuclei in blue (Hoechst). Scale bars 25 μm. b) 3D Deconvolution confocal images. (Left) Gastric organoids with normal inner polarity and large lumen. Immunofluorescence panel zonula occludens-1 (ZO-1) in violet, β-4 integrin (β-4 INT) in red, and nuclei in blue (Hoechst). Scale bars 50 μm. (Right) Gastric organoids with reverse polarity, showing an almost absent lumen. Immunofluorescence panel showing f-actin (F-ACT) in red, zonula occludens-1 (ZO-1) in violet, β-4 integrin (β-4 INT) in red, mucin 5AC (MUC5AC) in green and nuclei in blue (Hoechst). Scale bars 50 μm. c) Principal Component Analysis (PCA) of RNA-sequencing samples from RP-GOs at different stages of development (PCW 11, PCW 20, pediatric).

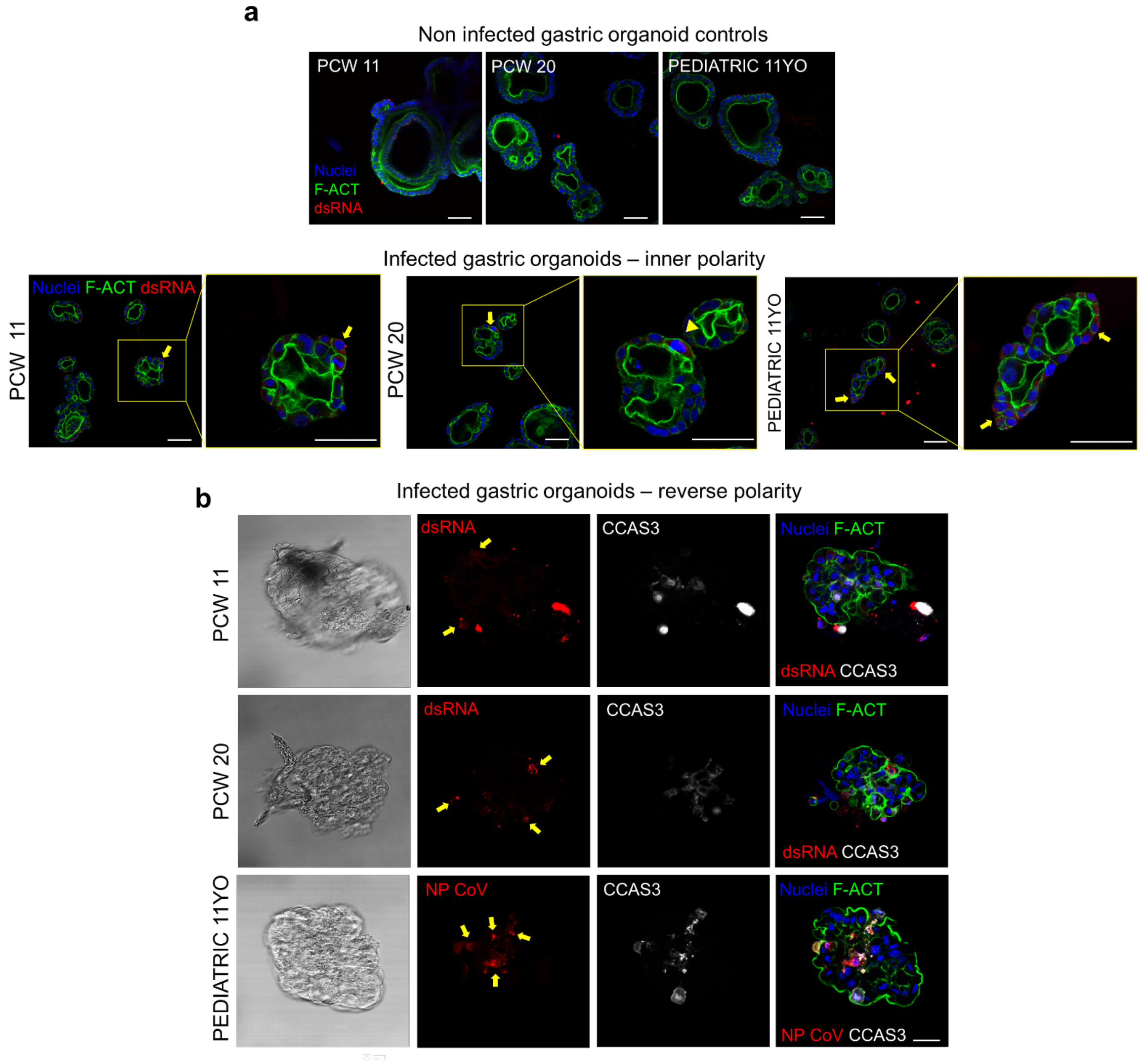

Supplementary Figure 4. SARS-CoV-2 staining at 64 h post-infection. a) Non-infected controls on Matrigel: whole organoids with normal polarity, showing absence of viral double strand RNA J2 (dsRNA) in red, f-actin (F-ACT) in green and nuclei in blue (Hoechst). Scale bars 50 μm (above). Low-efficiency infection in Matrigel whole organoids with normal polarity, showing viral double strand RNA J2 (dsRNA) in red, f-actin (F-ACT) in green and nuclei in blue (Hoechst). Scale bars 50 μm (above). b) SARS-CoV-2 staining at 96 h post-infection in reverse polarity gastric organoids. Immunofluorescence panel showing viral double strand RNA J2 (dsRNA) in red, SARS-CoV-2 Nucleocapsid Antibody (NP CoV) in red, cleaved caspase 3 (CCAS3) in white, f-actin (F-ACT) in green and nuclei in blue (Hoechst). Scale bar 50 μm.

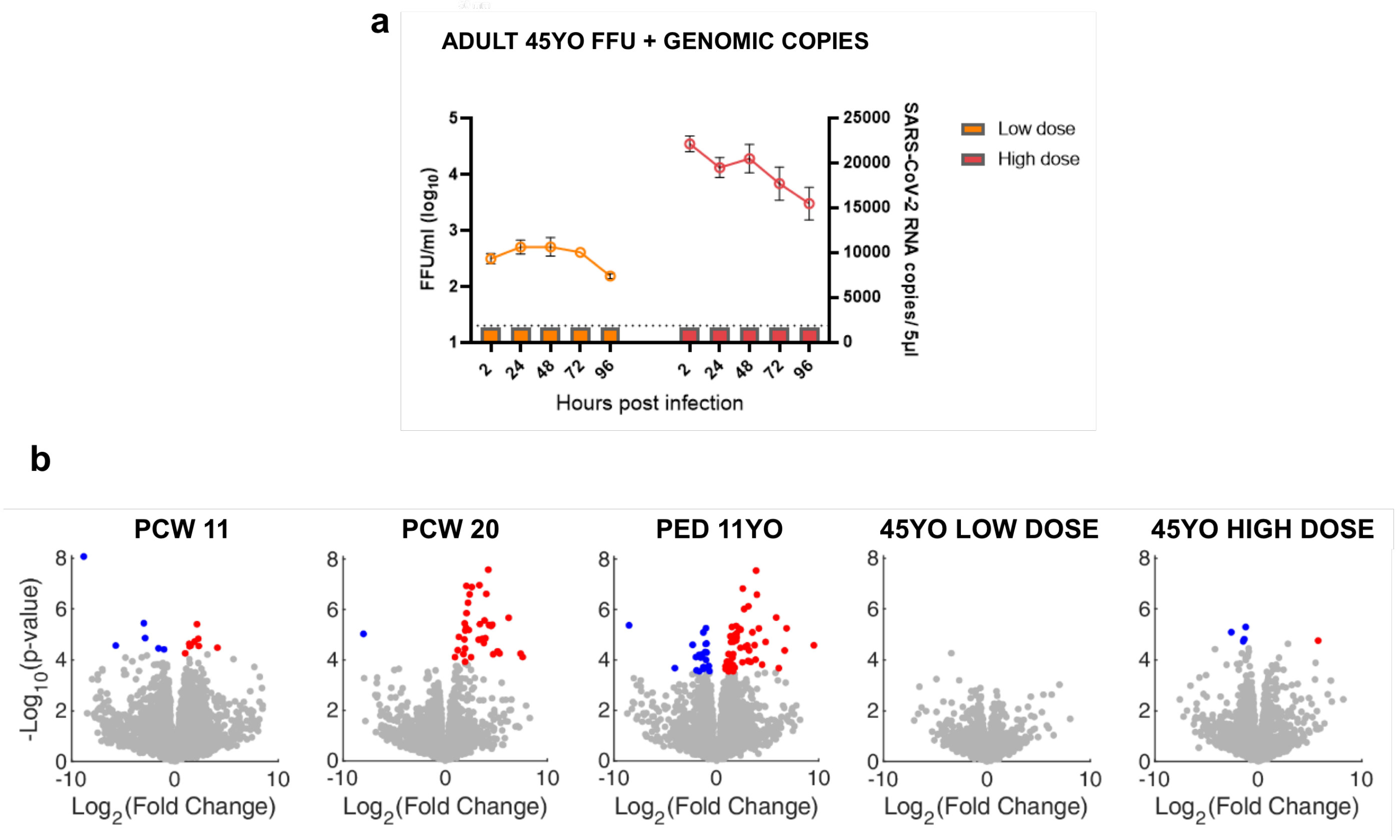

Supplementary Figure 5. a) Graph of SARS-CoV-2 replication in adult RP-GOs, showing Focus Forming Units (FFU) and genomic viral RNA copies, in low (MOI of 0.5) and high (MOI of 5) dose of infection. The dotted line indicates the lower limit of detection. Mean ± SD (N=3). b) Volcano plots indicating DEG genes of non-infected vs. infected sample comparisons at each developmental stage (PCW 11, PCW 20, 11-year pediatric, 45-year adult, and 45-year adult infected at higher dose with MOI of 5).

Supplementary Tables

Supplementary Table 1. Gastric samples list. Samples from which organoid lines were derived are highlighted in green.

Sample	Stage	Age	Use
1	Early fetal	CS20	Immuno
2	Early fetal	CS23	Macro/Immunofluorescence/Sequencing/RTPCR/Cell count
3	Early fetal	PCW 8 (late)	Macro
4	Early fetal	PCW 9	Cell count
5	Early fetal	PCW 9	Macro
6	Early fetal	PCW 10	Macro
7	Early fetal	PCW 10	Sequencing
8	Early fetal	PCW 11	Sequencing/RTPCR/Cell count/Immunofluorescence/Infection
9	Early fetal	PCW 11	Exapnsion
10	Early fetal	PCW 11	Cell count
11	Early fetal	PCW 11	Macro/Immunofluorescence/H&E
12	Early fetal	PCW 11	Macro/Immunofluorescence/H&E
13	Early fetal	PCW 11	Sequencing
14	Early fetal	PCW 12	Macro/Immunofluorescence/H&E
15	Early fetal	PCW 12	Macro/Immunofluorescence/H&E
16	Early fetal	PCW 12	Macro/Immunofluorescence/H&E
17	Early fetal	PCW 13	Macro/Immunofluorescence/H&E
18	Early fetal	PCW 14	Macro
19	Early fetal	PCW 15	Macro
20	Early fetal	PCW 15	Sequencing
21	Late Fetal	PCW 16	Macro/Immunofluorescence/H&E
22	Late Fetal	PCW 16	Macro/Immunofluorescence/H&E
23	Late Fetal	PCW 17	Macro/H&E
24	Late Fetal	PCW 17	Macro/H&E
25	Late Fetal	PCW 17	Macro/H&E
26	Late Fetal	PCW 17	Sequencing
27	Late Fetal	PCW 18	Sequencing/RTPCR/Cell count
28	Late Fetal	PCW 18	Macro/H&E
29	Late Fetal	PCW 18	Sequencing
30	Late Fetal	PCW 19	Cell count
31	Late Fetal	PCW 19	Macro
32	Late Fetal	PCW 19	Macro
33	Late Fetal	PCW 20	Sequencing/RTPCR/Cell count/Immunofluorescence/Infection
34	Late Fetal	PCW 20	Macro/Immunofluorescence/H&E
35	Late Fetal	PCW 20	Macro/Immunofluorescence/H&E
36	Late Fetal	PCW 20	Macro/Immunofluorescence/H&E
37	Late Fetal	PCW 20	Sequencing
38	Late Fetal	PCW 21	Macro/Immunofluorescence
39	Late Fetal	PCW 21	Macro/Immunofluorescence/Cell count
40	Pediatric	11 yo	Sequencing/RTPCR/Cell count/Immunofluorescence/Infection
41	Pediatric	1 yo	Sequencing/RTPCR
42	Pediatric	11 mo	Expansion
43	Pediatric	4 mo	Expansion
44	Pediatric	8 yo	Sequencing
45	Pediatric	8 yo	Sequencing
46	Pediatric	3 yo	Sequencing
47	Pediatric	8 yo	Sequencing
48	Pediatric	4 yo	Sequencing
49	Pediatric	13 yo	Sequencing
50	Pediatric	9 yo	Immunofluorescence
51	Pediatric	8 yo	Immunofluorescence
52	Pediatric	2 yo	Immunofluorescence
53	Pediatric	8 yo	Immunofluorescence
54	Pediatric	17 yo	Immunofluorescence
55	Adult	45 yo	Immunofluorescence/Infection/Sequencing
56	Adult	71 yo	Immunofluorescence/Infection

Supplementary Table 2. Human fetal and pediatric gastric organoid medium

Component	Stock conc.	Final conc.
Advanced DMEM F-12 (Thermo 12634)	-	To volume
HEPES (Thermo 15630080)	1 M	10 mM
Glutamax (Thermo 35050061)	100 X	2 mM
Pen/Strep (Thermo 15140122)	100 %	1 %
Primocin (Thermo Fisher NC9392943)	50 mg/mL	100 μg/mL
B-27 supplement minus vitamin A (Thermo 12587010)	50 X	1 X
n-acetylcysteine (Sigma A9165)	500 mM	1.25 mM
Wnt-3A (Peprotech 315-20)	50 μg/mL	100 ng/mL
R-spondin 1 (Peprotech 120-38)	100 μg/mL	500 ng/mL
Noggin (Peprotech 120-10C)	100 μg/mL	100 ng/mL
EGF (Thermo Fisher PMG8043)	500 μg/mL	50 ng/mL
Gastrin (Sigma G9020)	100 μM	10 nM
GSK-3 inhibitor (CHIR 99021) (Tocris 4423)	3 mM	3 μM
TGFb inhibitor (A83-01) (Sigma SML0788)	500 μM	5 μM
FGF10 (Peprotech 100-26)	100 μg/mL	200 ng/mL
ROCK inhibitor Y-27632 (Tocris 1254)	10 mg/mL	10 μg/mL

Supplementary Table 3. Antibody list

Antibody	Code	Brand	Host	Dilution
Chromogranin A (CHGA)	ab15160	Abcam	Rabbit	1:50
Mucin 5AC (MUC5AC)	ma5-12178	Invitrogen	Mouse	1:100
Mucin 6 (MUC6)	ab216017	Abcam	Mouse	1:100
Pepsinogen (PGC)	HPA031717	Atlas	Rabbit	1:100
Ezrin (EZR)	PA5-29358	Thermo Fisher	Rabbit	1:200
Zonula occludens-1 (ZO-1)	40-2200	Life Technologies	Rabbit	1:200
Somatostatin (SST)	MAB2358	R&D Systems	Rat	1:100
Integrin beta-4 (INTB4)	ab110167	Abcam	Rat	1:200
Cleaved Caspase-3 (CCAS3)	9661	Cell Signaling	Rabbit	1:400
TMPRSS2	MABF2158	Sigma-Aldrich	Mouse	1:100
Angiotensin-converting enzyme 2 (ACE2)	AF933	R&D Systems	Goat	1:100
Angiotensin-converting enzyme 2 (ACE2)	MAB933	R&D Systems	Mouse	1:100
Double Strand RNA (dsRNA J2)	J2	Scicons	Mouse	1:200
SARS-CoV/SARS-CoV-2 Nucleocapsid (NP CoV)	40143-MM05	Sino Biological	Mouse	1:200
anti-Mouse 647 (secondary AB)	A31571	Thermo Fisher	Donkey	1:300
anti-Rabbit 647 (secondary AB)	A21244	Thermo Fisher	Goat	1:300
anti-Rat 594 (secondary AB)	A11007	Thermo Fisher	Goat	1:300
anti-Rabbit 594 (secondary AB)	A11012	Thermo Fisher	Goat	1:300
anti-Rabbit 568 (secondary AB)	A11042	Thermo Fisher	Donkey	1:300
anti-Rabbit 488 (secondary AB)	A11008	Thermo Fisher	Goat	1:300
anti-Mouse 488 (secondary AB)	A11001	Thermo Fisher	Goat	1:300
anti-Mouse 568 (secondary AB)	A10037	Thermo Fisher	Goat	1:300
Phalloidin (f-actin)	A22287	Thermo Fisher	---	1:100
Phalloidin (f-actin)	A12379	Thermo Fisher	---	1:100
Hoechst 33342 (nuclear staining)	H1399	Thermo Fisher	---	10 μg/mL
