# Supplementary material for: SARS-CoV-2 infection and replication in human gastric organoids
Source: Nat Commun. 2021 Nov 16;12:6610. doi: 10.1038/s41467-021-26762-2 (PMC8595698; doi:10.1038/s41467-021-26762-2)
Supplement: Supplementary file 2 — Reporting Summary [file 41467_2021_26762_MOESM2_ESM.pdf]

nature portfolio

Corresponding author(s): Paolo De Coppi; Nicola Elvassore; Giovanni Giuseppe Giobbe

Last updated by author(s): Sep 9, 2021

Reporting Summary

Nature Portfolio wishes to improve the reproducibility of the work that we publish. This form provides structure for consistency and transparency in reporting. For further information on Nature Portfolio policies, see our Editorial Policies and the Editorial Policy Checklist.

Statistics

For all statistical analyses, confirm that the following items are present in the figure legend, table legend, main text, or Methods section.

n/a	Confirmed	
☐	☒	The exact sample size (*n*) for each experimental group/condition, given as a discrete number and unit of measurement
☐	☒	A statement on whether measurements were taken from distinct samples or whether the same sample was measured repeatedly
☐	☒	The statistical test(s) used AND whether they are one- or two-sided<br>*Only common tests should be described solely by name; describe more complex techniques in the Methods section.*
☒	☐	A description of all covariates tested
☒	☐	A description of any assumptions or corrections, such as tests of normality and adjustment for multiple comparisons
☐	☒	A full description of the statistical parameters including central tendency (e.g. means) or other basic estimates (e.g. regression coefficient) AND variation (e.g. standard deviation) or associated estimates of uncertainty (e.g. confidence intervals)
☐	☒	For null hypothesis testing, the test statistic (e.g. *F*, *t*, *r*) with confidence intervals, effect sizes, degrees of freedom and *P* value noted<br>*Give P values as exact values whenever suitable.*
☒	☐	For Bayesian analysis, information on the choice of priors and Markov chain Monte Carlo settings
☐	☒	For hierarchical and complex designs, identification of the appropriate level for tests and full reporting of outcomes
☒	☐	Estimates of effect sizes (e.g. Cohen's *d*, Pearson's *r*), indicating how they were calculated

Our web collection on statistics for biologists contains articles on many of the points above.

Software and code

Policy information about availability of computer code

Data collection

For RNA-seq data of original tissues and organoids with spontaneous polarity, total RNA (100 ng) from each sample was prepared using QuantSeq 3' mRNA-Seq Library prep kit (Lexogen GmbH) according to manufacturer's instructions. The amplified fragmented cDNA of 300 bp in size were sequenced in single-end mode using the Nova Seq 6000 (Illumina) with a read length of 100 bp. Illumina novaSeq base call (BCL) files were converted into fastq files through bcl2fastq (version v2.20.0.422) following software guide. Sequence reads were trimmed using bbduk software (bbmap suite 37.31), following software guide, to remove adapter sequences, poly-A tails and low-quality end bases (regions with average quality below 6). Alignment was performed with STAR 2.6.0a44 on hg38 reference assembly obtained from cellRanger website (Ensembl 93), following online site guide. The expression levels of genes were determined with htseq-count 0.9.1 by using cellRanger pre-build genes annotations (Ensembl Assembly 93). All transcripts having <1 CPM in less than 4 samples and percentage of multimap alignment reads > 20% simultaneously were filtered out.

For RNA-seq data of non-infected and infected RP-GOs, a total of 600 pg of RNA was used as input for the synthesis of cDNA with the SMART-Seq v4 Ultra Low Input RNA Kit for Sequencing (Takara Bio USA, Mountain View, CA, USA). Manufacturer suggested protocol was followed, with minor modifications. 75 pg of DNA generated with SMART-Seq v4 Kit were used for preparation of library with NEXTERA XT DNA Library Preparation kit (Illumina Inc., San Diego, CA, USA), following suggested protocol. Libraries were sequenced in pair-end mode using a Nova Seq 6000 sequencing system on an SP, 100 cycles flow cell (Illumina Inc., San Diego, CA, USA). Illumina novaSeq base call (BCL) files were converted into fastq files through bcl2fastq (version v2.20.0.422) following software guide. Alignment was performed with STAR 2.6.0a44 on hg38 reference assembly obtained from the Gencode website (primary assembly v. 32). Transcripts estimated counts were determined with RSEM 1.3.045 by using the Gencode v.32 genes annotations. All genes having <1 CPM in less than 2 replicates of the same condition were filtered out.

Data analysis

Statistical analyses were performed using the following software: MATLAB (v. R2017a) for PCA, pie plot, bar plot, hierarchical clustering with proteomic and RNA-seq data. Graph Pad Prism Mac (v. 6.0h) was used with all other graphs and charts. Numerosity of representative

experiments: Fig. 1c-e representative of n = 3. Fig. 2a-b representative of n = 3. Fig. 2c and Suppl. Fig. 3a representative of n = 50 stained organoids per line. Fig. 4b-c representative of 6 well plates full of organoids, with each polarity reversion experiment repeated n = 8 for each cell line. Fig. 4f representative of n = 3 tissue slides per sample. Fig. 4g representative of n = 50 stained organoids per line. Fig. 5a representative of n > 100 organoids per line. Fig. 5b-c and Suppl. Fig. 4a-b representative of n = 50 stained organoids per line. Fig. 6a representative of n = 20 stained organoids per line. All other numerosity and statistical tests used are reported in the figure legends.

For RNA sequencing data analyses, differentially expressed genes (DEGs) were computed in R (version 3.5) with edgeR (version 3.24)46, using a mixed criterion based on p-value, after false discovery rate (FDR) correction by Benjamini-Hochberg method, lower than 0.05 and absolute log2(fold change) higher than log2(1.5). This analysis was paired between non-infected and infected samples derived from the same original sample. For RNA-seq data of organoids with spontaneous polarity, DEGs were clustered according to a flat, increasing, or decreasing profile according to the differential expression analysis between pairs of time points. Principal Component Analysis was performed by Singular Value Decomposition (SVD) on log2(CPM+1) data, after centering, using MATLAB R2017a (The MathWorks). DEGs over-representation analysis of Gene Ontology (GO, https://www.ebi.ac.uk/GOA) and Reactome (https://reactome.org/) categories was performed using ClueGO (version 2.5.4)47. Reactome hierarchy was visualized using ClueGO within Cytoscape (version 3.5)48. Hierarchical clustering of DEGs was performed on median-centered log2(CPM+1) data in MATLAB, using Euclidean distance and complete linkage. Log-normalized expression data were analyzed by the Quantitative Set Analysis for Gene Expression (QuSAGE, version 2.16)33 Bioconductor (version 3.8) package.

For manuscripts utilizing custom algorithms or software that are central to the research but not yet described in published literature, software must be made available to editors and reviewers. We strongly encourage code deposition in a community repository (e.g. GitHub). See the Nature Portfolio guidelines for submitting code & software for further information.

Data

Policy information about availability of data

All manuscripts must include a data availability statement. This statement should provide the following information, where applicable:

- Accession codes, unique identifiers, or web links for publicly available datasets
- A description of any restrictions on data availability
- For clinical datasets or third party data, please ensure that the statement adheres to our policy

The authors declare that all data supporting the findings of this study are available within the article, its Supplementary Information, Source Data files, and online deposited data. The Gastric QuantSeq RNA-seq data generated in this study, presented in Fig. 3a-f, Fig. 4d, and Supplementary Fig. 2a-g have been deposited in the NCBI GEO database under accession code GSE153698: https://www-ncbi-nlm-nih-gov.ezproxy.u-pec.fr/geo/browse/?view=samples&series=153698. The Gastric SMART RNA-seq data presented in Fig. 4e, Fig. 5e-h, Supplementary Fig. 3c, and Supplementary Fig. 5b have been deposited in the NCBI GEO database under accession code GSE153684: https://www.ncbi.nlm.nih.gov/geo/query/acc.cgi?acc=GSE153684. The Gastric SMART RNA-seq data presented in Fig. 4e, and Supplementary Fig. 5b have been deposited in the NCBI GEO database under accession code GSE184390: https://www.ncbi.nlm.nih.gov/geo/query/acc.cgi?acc=GSE184390.

Field-specific reporting

Please select the one below that is the best fit for your research. If you are not sure, read the appropriate sections before making your selection.

[x] Life sciences   [ ] Behavioural & social sciences   [ ] Ecological, evolutionary & environmental sciences

For a reference copy of the document with all sections, see nature.com/documents/nr-reporting-summary-flat.pdf

Life sciences study design

All studies must disclose on these points even when the disclosure is negative.

Sample size

Sample size for every experiment performed and image acquired is presented in the figure legends and in the "Statistics and Reproducibility" paragraph.

For imaging data, images in the manuscript are representative images of a minimum n=3 experiments, with sample size determined by the availability of patient samples.

For quantitative analyses, no sample size calculation was performed but the sample size / replicate number was chosen in order to provide sufficient data points for the determination of measures of central tendency, variance, and parametric vs non-parametric distribution of the data.

Statistical significance of reported results was assessed by statistical tests during data analyses, as indicated in Methods section.

Data exclusions

No data were excluded from the analyses.

Replication

Cell culture experiments were replicated within our laboratory by at least two independent operators (G.G.G and B.C.J), and independently across laboratories and Universities (UCL and University of Padova), a minimum of n=3 times per experiment. Infection experiments were performed in the infection facilities at the University of Padova and repeated a minimum of n=5 times with each cell line.

All attempts at replication were successful, other than for the initial infection experiments on adult gastric organoids as fully described in the results section of the manuscript.

Randomization

Randomization was not required in this study because the study design intentionally compared experimental groups that were defined based

	on know differences between their developmental stage and age. This in vitro study includes appropriate positive and negative controls, as presented in the manuscript.
Blinding	Immunofluorescence of infected organoids were analysed in blind and with same settings for controls and samples. The investigator analyzing the experiment was not the same investigator performing the experiment for all immunofluorescence and qPCR data. During data collection, samples were given codes to blind the analyzing investigator from group allocation. RNAseq analysis was done by the bioinfomatician independent of the laboratory investigators, who received the data already blinded to conceal their group allocations. Blinding was performed in a first stage of the data analysis, then it was not possible due to participants involvement in data discussion.

Reporting for specific materials, systems and methods

We require information from authors about some types of materials, experimental systems and methods used in many studies. Here, indicate whether each material, system or method listed is relevant to your study. If you are not sure if a list item applies to your research, read the appropriate section before selecting a response.

Materials & experimental systems

n/a	Involved in the study
☐	☒ Antibodies
☐	☒ Eukaryotic cell lines
☒	☐ Palaeontology and archaeology
☒	☐ Animals and other organisms
☐	☒ Human research participants
☒	☐ Clinical data
☒	☐ Dual use research of concern

Methods

n/a	Involved in the study
☒	☐ ChIP-seq
☒	☐ Flow cytometry
☒	☐ MRI-based neuroimaging

Antibodies

Antibodies used

Antibody, Supplier, Catalog Number, Clone Name, Lot Number, Host, Dilution
Chromogranin A (CHGA), Abcam, ab15160, polyclonal, GR3190950-1, Rabbit, 1:50
Mucin 5AC (MUC5AC), Invitrogen, MA5-12178, 45M1, RL2306615, Mouse, 1:100
Mucin 6 (MUC6), Abcam, ab216017, MUC6/916, GR310618-9, Mouse, 1:100
Pepsinogen (PGC), Atlas Antibodies, HPA031717, polyclonal, A106241, Rabbit, 1:100
Ezrin (EZR), Invitrogen, PA5-29358, polyclonal, SB23819310, Rabbit, 1:200
Zonula occludens-1 (ZO-1), Invitrogen, 40-2200, polyclonal, SG246825, Rabbit, 1:200
Integrin beta-4 (INTB4), Abcam, ab110167, 439-9B, GR3385737-1, Rat, 1:200
Somatostatin (SST), R&D Systems, MAB2358, 906552, CIVM0319071, Rat, 1:100
Cleaved Caspase-3 (CCAS3), Cell Signaling Technology, 9661, D175, 45, Rabbit, 1:400
Transmembrane protease serine 2 (TMPRSS2), EMD Millipore, MABF2158, P5H9-A3, 3474105, Mouse, 1:100
Angiotensin-converting enzyme 2 (ACE2), R&D Systems, AF933, polyclonal, HOK0320041, Goat, 1:100
Angiotensin-converting enzyme 2 (ACE2), R&D Systems, MAB933, 171696, HYA0320041, Mouse, 1:100
Double Strand RNA (dsRNA), Scicons, 10010500, J2, Mouse, 1:200
SARS-CoV/SARS-CoV-2 Nucleocapsid (NP CoV), Sino Biological, 40143-MM05, IgG1 Clone #05, HB14MY2001, Mouse, 1:200
anti-Rabbit 488 (secondary AB), Invitrogen, A11008, IgG H+L, 1981125, Goat, 1:300
anti-Mouse 488 (secondary AB), Invitrogen, A11001, IgG H+L, 1810918, Goat, 1:300
anti-Rabbit 568 (secondary AB), Invitrogen, A11042, IgG H+L, 2207536, Donkey, 1:300
anti-Mouse 568 (secondary AB), Invitrogen, A10037, IgG H+L, 1696197, Goat, 1:300
anti-Rat 594 (secondary AB), Invitrogen, A11007, IgG H+L, 1008647, Goat, 1:300
anti-Rabbit 594 (secondary AB), Invitrogen, A11012, IgG H+L, 1892265, Goat, 1:300
anti-Mouse 647 (secondary AB), Invitrogen, A31571, IgG H+L, 1900251, Donkey, 1:300
anti-Rabbit 647 (secondary AB), Invitrogen, A21244, IgG H+L, 1246457, Goat, 1:300
Phalloidin 488 (f-actin), Invitrogen, A12379, n/a, 1656101, n/a, 1:100
Phalloidin 647 (f-actin), Invitrogen, A22287, n/a, 2015553, n/a, 1:100
Hoechst 33342 (nuclear staining), Invitrogen, H1399, n/a, 1932847, n/a, 10 µg/mL

Validation

Antibodies were validated on primary gastric tissue and using validation information on the manufacturer's website. Links to validation information from the manufacturers are included here:

Chromogranin A (CHGA), Abcam, ab15160, https://www.abcam.com/chromogranin-a-antibody-ab15160.html
Mucin 5AC (MUC5AC), Invitrogen, MA5-12178, https://www.thermofisher.com/antibody/product/MUC5AC-Antibody-clone-45M1-Monoclonal/MA5-12178
Mucin 6 (MUC6), Abcam, ab216017, https://www.abcam.com/gastric-mucinmuc-6-antibody-muc6916-ab216017.html
Pepsinogen (PGC), Atlas Antibodies, HPA031717, https://www.atlasantibodies.com/products/antibodies/primary-antibodies/triple-a-polyclonals/pgc-antibody-hpa031717/
Ezrin (EZR), Invitrogen, PA5-29358, https://www.thermofisher.com/antibody/product/Ezrin-Antibody-Polyclonal/PA5-29358
Zonula occludens-1 (ZO-1), Invitrogen, 40-2200, https://www.thermofisher.com/antibody/product/ZO-1-Antibody-Polyclonal/40-2200

Integrin beta-4 (INTB4), Abcam, ab110167, https://www.abcam.com/integrin-beta-4-antibody-439-9b-ab110167.html
Somatostatin (SST), R&D Systems, MAB2358, https://www.rndsystems.com/products/human-mouse-somatostatin-antibody-906552_mab2358
Cleaved Caspase-3 (CCAS3), Cell Signaling Technology, 9661, https://www.cellsignal.co.uk/products/primary-antibodies/cleaved-caspase-3-asp175-antibody/9661
Transmembrane protease serine 2 (TMPRSS2), EMD Millipore, MABF2158, https://www.merckmillipore.com/GB/en/product/Anti-TMPRSS2-Antibody-clone-P5H9-A3,MM_NF-MABF2158-100UG
Angiotensin-converting enzyme 2 (ACE2), R&D Systems, AF933, https://www.rndsystems.com/products/human-mouse-rat-hamster-ace-2-antibody_af933
Angiotensin-converting enzyme 2 (ACE2), R&D Systems, MAB933, https://www.rndsystems.com/products/human-hamster-ace-2-antibody-171606_mab933
Double Strand RNA (dsRNA), Scicons, 10010500, https://www.nordicmubio.com/products/product/10010500
SARS-CoV/SARS-CoV-2 Nucleocapsid (NP CoV), Sino Biological, 40143-MM05, https://www.sinobiological.com/antibodies/cov-nucleocapsid-40143-mm05
anti-Rabbit 488 (secondary AB), Invitrogen, A11008, https://www.thermofisher.com/antibody/product/Goat-anti-Rabbit-IgG-H-L-Cross-Adsorbed-Secondary-Antibody-Polyclonal/A-11008
anti-Mouse 488 (secondary AB), Invitrogen, A11001, https://www.thermofisher.com/antibody/product/Goat-anti-Mouse-IgG-H-L-Cross-Adsorbed-Secondary-Antibody-Polyclonal/A-11001
anti-Rabbit 568 (secondary AB), Invitrogen, A10042, https://www.thermofisher.com/antibody/product/A10042.html?ef_id=Cj0KCQjw4eaJBhDMARIsANhrQAAUQ-pcZPdEBXDD_9FresnQDE5kwQW4XhccNE0Vd9GlqHpTsAEjSVgaAuY4EALw_wcB:G:s&s_kwcid=AL!3652!3!516608152221!b!!g!!&cid=bid_pca_aus_r01_co_cp1359_pjt0000_bid00000_0se_gaw_dy_pur_con&gclid=Cj0KCQjw4eaJBhDMARIsANhrQAAUQ-pcZPdEBXDD_9FresnQDE5kwQW4XhccNE0Vd9GlqHpTsAEjSVgaAuY4EALw_wcB
anti-Mouse 568 (secondary AB), Invitrogen, A10037, https://www.thermofisher.com/antibody/product/Donkey-anti-Mouse-IgG-H-L-Highly-Cross-Adsorbed-Secondary-Antibody-Polyclonal/A10037
anti-Rat 594 (secondary AB), Invitrogen, A11007, https://www.thermofisher.com/antibody/product/Goat-anti-Rat-IgG-H-L-Cross-Adsorbed-Secondary-Antibody-Polyclonal/A-11007
anti-Rabbit 594 (secondary AB), Invitrogen, A11012, https://www.thermofisher.com/antibody/product/Goat-anti-Rabbit-IgG-H-L-Cross-Adsorbed-Secondary-Antibody-Polyclonal/A-11012
anti-Mouse 647 (secondary AB), Invitrogen, A31571, https://www.thermofisher.com/antibody/product/Donkey-anti-Mouse-IgG-H-L-Highly-Cross-Adsorbed-Secondary-Antibody-Polyclonal/A-31571
anti-Rabbit 647 (secondary AB), Invitrogen, A21244, https://www.thermofisher.com/antibody/product/Goat-anti-Rabbit-IgG-H-L-Cross-Adsorbed-Secondary-Antibody-Polyclonal/A-21244
Phalloidin 488 (f-actin), Invitrogen, A12379, https://www.thermofisher.com/order/catalog/product/A12379#/A12379
Phalloidin 647 (f-actin), Invitrogen, A22287, https://www.thermofisher.com/order/catalog/product/A22287#/A22287
Hoechst 33342 (nuclear staining), Invitrogen, H1399, https://www.thermofisher.com/order/catalog/product/H1399#/H1399

Subsequent staining protocols included positive and negative controls.

Eukaryotic cell lines

Policy information about cell lines

Cell line source(s)	Human fetal cells were isolated from gastric tissue obtained immediately after termination of pregnancy from 8 to 21 PCW (post conception week).<br>Human pediatric and adult cells were isolated from gastric surgical biopsies and endoscopies immediately after sample collection.<br><br>Vero E6 cells were purchased from ATCC: VERO C1008 [Vero 76, clone E6, Vero E6] CRL-1586™
Authentication	Organoid lines were not authenticated.<br><br>Vero E6 are autenticated by the producing company and commercially available: "This line is a clone of VERO 76 (ATCC CRL-1587). It was cloned by the dilution method into microtiter plates in 1979 by P.J. Price.- Plaques are also produced."
Mycoplasma contamination	All cell lines used in this work were tested monthly and always tested negative for Mycoplasma. Vero E6 is guarantedd mycoplasma tested negative by the company.
Commonly misidentified lines<br>(See ICLAC register)	Not relevant

Human research participants

Policy information about studies involving human research participants

Population characteristics	Fetal samples from 8 to 21 PCW (post conception week) fetuses.<br>Pediatric samples were collected from 4 months-old to 17 years-old patients.<br>Adult samples were collected from 45 and 71 years-old patients.<br>Full list of samples is provided in Supplementary Table 1.
Recruitment	Fetal samples were sourced following patient informed consent via the Joint MRC/Wellcome Trust Human Developmental Biology Resource with Research Tissue Bank ethical approval.<br><br>Human pediatric and adult gastric surgical biopsies were collected after patient informed consent, in compliance with all relevant ethical regulations for work with human participants.<br><br>Informed consent was undertaken by an independent research coordinator from the BRC. The authors were not involved in any phase of the consent process and every patient (or their parent or person with parental responsibility, for minors) undergoing gastric surgery or upper gastrointestinal endoscopy at Great Ormond Street Hospital was approached by the research coordinator for involvement in the study. Adult gastric organoid lines were obtained by Material Transfer Agreement from the Hubrecht Institute in the Netherlands.<br><br>The SARS-CoV-2 isolate was obtained from a nasopharyngeal swab collected from a 14-year-old boy during routine diagnostic activities conducted at the University Hospital of Padua (Italy). Legally authorized representatives of the subject provided written consent for the collection and use of biological specimens for research purposes
Ethics oversight	Fetal samples: Joint MRC/Wellcome Trust Human Developmental Biology Resource under informed ethical consent with Research Tissue Bank ethical approval (08/H0712/34+5).<br><br>Pediatric samples: NHS Health Research Authority - East of England – Cambridge Central Research Ethics Committee (NHS licenses 08ND13 and 18DS02)<br><br>Adult organoids were obtained through material transfer agreement and were previously derived in the Hubrecht Institute under a study approved by the ethical committee of the University Medical Center Utrecht (UMCU; the Netherlands) and in accordance with the Declaration of Helsinki. Patients gave informed consent in compliance to Dutch law and all relevant ethical regulations regarding research involving human participants.<br><br>Virus sample: Protocol N° 0070714; amendment number 71779; Ethics Committee of the Azienda Ospedale Università di Padova

Note that full information on the approval of the study protocol must also be provided in the manuscript.
