# Supplementary material for: SARS-CoV-2 infection and replication in human gastric organoids
Source: Nat Commun. 2021 Nov 16;12:6610. doi: 10.1038/s41467-021-26762-2 (PMC8595698; doi:10.1038/s41467-021-26762-2)
Supplement: Supplementary file 3 — Description of Additional Supplementary Files [file 41467_2021_26762_MOESM3_ESM.pdf]

Description of Additional Supplementary Files

File Name: Supplementary Data 1
Description: List of significantly differentially expressed genes progressively down-/up-regulated in samples derived from donors of increasing fetal and pediatric age, with similar trend in tissues and organoids. Only genes with up-up or down-down trend were included. Expression is reported as CPM.

File Name: Supplementary Data 2
Description: Full results of clusters enrichment analysis shown in Fig. 3f.

File Name: Supplementary Data 3
Description: Results from a Quantitative Set Analysis for Gene Expression (QuSAGE) within the Gene Ontology database for data shown in Fig. 5. Paired comparisons between non-infected and infected samples were performed at each developmental stage.
